# Ebola: translational science considerations

**DOI:** 10.1186/s12967-014-0362-3

**Published:** 2015-01-16

**Authors:** Francesco Chiappelli, Andre Bakhordarian, April D Thames, Angela M Du, Allison L Jan, Melissa Nahcivan, Mia T Nguyen, Nateli Sama, Ercolano Manfrini, Francesco Piva, Rafael Malagoli Rocha, Carl A Maida

**Affiliations:** UCLA School of Dentistry (Oral Biology & Medicine), Los Angeles, USA; Evidence-Based Decision Practice-Based Research Network, Los Angeles, USA; UCLA David Geffen School of Medicine (Psychiatry), Los Angeles, USA; Regional Health District Marche Ancona Hospital, Ancona, Italy; Polytechnic University of the Marche Region (Odontostomatological Sciences), Ancona, Italy; A C Camargo Cancer Center, Sao Paulo, Brazil; UCLA School of Dentistry (Public Health Dentistry), UCLA Institute of the Environment and Sustainability, UCLA Center for Tropical Research, Los Angeles, USA; UCLA Center for the Health Sciences 63-090, 10833 Le Conte Avenue, Los Angeles, CA 90095-1668 USA

**Keywords:** Zaire Ebola virus, Ebola virus disease, Cell-mediated immune surveillance, Vaccine, Blood–brain barrier, M1 & M2 macrophages, Epidemic, Pandemic, Syndemic, Zoonotic

## Abstract

We are currently in the midst of the most aggressive and fulminating outbreak of Ebola-related disease, commonly referred to as “Ebola”, ever recorded. In less than a year, the Ebola virus (EBOV, Zaire ebolavirus species) has infected over 10,000 people, indiscriminately of gender or age, with a fatality rate of about 50%. Whereas at its onset this Ebola outbreak was limited to three countries in West Africa (Guinea, where it was first reported in late March 2014, Liberia, where it has been most rampant in its capital city, Monrovia and other metropolitan cities, and Sierra Leone), cases were later reported in Nigeria, Mali and Senegal, as well as in Western Europe (i.e., Madrid, Spain) and the US (i.e., Dallas, Texas; New York City) by late October 2014. World and US health agencies declared that the current Ebola virus disease (EVD) outbreak has a strong likelihood of growing exponentially across the world before an effective vaccine, treatment or cure can be developed, tested, validated and distributed widely. In the meantime, the spread of the disease may rapidly evolve from an epidemics to a full-blown pandemic. The scientific and healthcare communities actively research and define an emerging kaleidoscope of knowledge about critical translational research parameters, including the virology of EBOV, the molecular biomarkers of the pathological manifestations of EVD, putative central nervous system involvement in EVD, and the cellular immune surveillance to EBOV, patient-centered anthropological and societal parameters of EVD, as well as translational effectiveness about novel putative patient-targeted vaccine and pharmaceutical interventions, which hold strong promise, if not hope, to curb this and future Ebola outbreaks. This work reviews and discusses the principal known facts about EBOV and EVD, and certain among the most interesting ongoing or future avenues of research in the field, including vaccination programs for the wild animal vectors of the virus and the disease from global translational science perspective.

## Introduction

On the 23rd of March 2014, the World Health Organization (WHO) was notified of an outbreak of Ebola virus disease (EVD), a type of aggressive hemorrhagic fever with a high average fatality rate, which had apparently begun three months earlier, in December 2013, in Méliandou a town of 12,000 people located in the *Préfecture de Guékédou*, N’zerekore Region of the *Guinée Forestière*, in the eastern sector of the Republic of Guinea, on the Atlantic coast of West Africa. Soon, cases were confirmed across Guinea, including in the Guinean capital, Conakry, as well as in neighboring Sierra Leone, and in the Republic of Liberia, including its capital Monrovia.

Previously known as Ebola hemorrhagic disease, EVD had until now only manifested in a few focal tropical regions of Sub-Saharan Africa. From 1976, when EVD was first identified, through 2013, WHO had reported in total fewer than 2,000 confirmed cases. But, as of early August 2014, the number of cases of the present epidemic had already surpassed the number of confirmed cases of the previous outbreaks combined. As of mid-Fall 2014, the number of deaths attributed to EVD was close to 5,000, including a fatality on US soil [1].

The efforts of WHO have been to contain and to prevent further spreading of EVD, while supporting at-risk countries to develop preparedness plans [1]. The Ebola virus (EBOV), the causative agent of EVD, is classified as a Category-A infectious substance by the U.S. Department of Transportation’s Hazardous Materials Regulations (HMR:49 C.F.R., Parts 171–180) [1,2].

Professor Peter Piot, now director of the London School of Hygiene and Tropical Medicine, and his team first identified Ebola, on the 26th of August 1976, in Yambuku, a small rural village in Mongala District of northern Democratic Republic of the Congo (then known as Zaire). The first victim, patient zero for EVD, was the village school headmaster Mabalo Lokela, who had toured an area near the Central African Republic border along the Ebola river between the 12th and the 22nd of August of that year. The virus responsible for Mr. Lokela’s initial infection was first thought to be Marburg virus (MBGV), but later identified as a new type of virus related to MGBV, and named after the nearby Ebola river. Two weeks after the first onset of symptoms, on the 8th of September, Mr. Lokela died of the disease resulting from this infection with the Ebola virus. The specific species of EBOV responsible for the first outbreak in the Democratic Republic of the Congo, now Zaire, was called the Zaire EBOV ebolavirus species [1].

Subsequently a number of additional cases were reported. Nearly all were centered in the Yambuku mission hospital, or were traced to individuals having had close contact with its patient and health care provider population. A total of 318 cases were diagnosed and 280 deaths were recorded, corresponding to an 88% fatality rate. A second EBOV species, Sudan EBOV, was identified that same year when EVD erupted in Sudan, and claimed 284 infected people, and 151 deaths (53% fatality rate) [1]. This new emerging fulminating contagious viral disease stunned the World health community.

These initial Ebola outbreaks were contained with the help of WHO, the Congolese air force that secured transport of necessary medical supplies, food and water, and by common-sense public health protocols, including quarantining villagers, sterilizing medical equipment, and providing protective clothing (i.e., personal protective equipment, PPE) [1].

EVD reappeared in the Democratic Republic of Congo two decades later, in 1995. With a fatality rate of 81%, this Zaire EBOV species outbreak affected 315, killing 254 people. This prompted the Infectious Diseases journal, published by the CDC, to produce its first Ebola article in its first volume (number 3).

Fifteen years later (2000), Uganda suffered a Sudan EBOV species outbreak, which targeted 425 people, killing 224 (53% fatality rate). Three years later (2003), the Zaire EBOV species struck Zaire again, infecting 143 people, with a 90% fatality rate (128 deaths).

Yet again in 2007, the Zaire EBOV species struck in the village of Kampungu of the Kasai-Occidental region of the Democratic Republic of the Congo, about 420 miles from Kinshasa. The disease was contained only after it had spread to four contiguous villages: in total, 264 individuals fell sick, with a fatality rate of 71% [1].

One key 2007 study [2] tested 54 specimens obtained from 26 patients of this 2007 outbreak, 12 of who had died of Ebola. The samples were collected, and stored in liquid nitrogen until a batch-reverse transcription polymerase chain reaction (RT-PCR) assay was performed. Sixteen clinical specimens from 12 patients were positive by virus culture (25% true positives) and RT-PCR (100% true positives). All samples of plasma and serum (16 of 16), tears (1 of 1), nasal blood (1 of 1) and breast milk (2 of 2) were positive for EBOV. Half of the samples of saliva (8 of 16) were positive by RT-PCR, as well as half of the stool and semen samples. None of the samples of *sputum*, sweat or vomit showed any trace of the virus by RT-PCR. The shedding of EBOV in blood and saliva corresponded almost exactly to the period of acute viremia and associated EVD symptomatology. Positive saliva specimens were obtained not before day 8 after disease onset. No sample was tested positive during the prodromic phase of the disease. Based on those preliminary pilot data, inferences were drawn that EBOV might not be contagious during the asymptomatic phase of infection, and that EBOV is found primarily in bodily fluid, and thus might be transmitted only thusly. In retrospect, however, this study appears now to have been grossly under-powered to warrant application of these inferences in the present outbreak.

Later that same year, on the 30th of November 2007, the Uganda Ministry of Health confirmed EVD in the Bundibugyo District in Western Uganda. The virus sample was tested and the findings, confirmed by the US National Reference Laboratories, CDC, and WHO, revealed the emergence of a third and new EBOV species, which was named Bundibugyo. That outbreak claimed 149 victims, of whom 37 were lethal cases (25% fatality rate) [1].

Five years later (2012), again in Uganda, the Sudan EBOV species struck twice, but both outbreaks were immediately contained with only seven people infected on the first and 24 on the second, and a fatality rate of 57% and 71% respectively. On the 17th of August 2012, the Ministry of Health of the Democratic Republic of the Congo reported Bundibugyo-EBOV disease in the eastern region, about the towns of Isiro and Viadana. Of 57 people who fell sick, 29 perished (51% fatality rate) [1].

The current fulminating outbreak (Zaire EBOV species) was first reported a year and a half later, on the 23rd of March 2014 in Guinea, West Africa. Patient zero was identified to be a two-year old child who had died on the 28th of December 2013. Professor Piot was quoted as saying that “…*actually … this outbreak was dying out in May, in Guinea. Then a famous woman, a traditional healer, died, and at her funeral hundreds of people touched the body. Then there was this explosion in three countries*…” (The Telegraph, 19 October 2014). Since then, the disease has spread rapidly to the neighboring West African countries of Liberia and Sierra Leone. Contrary to the previous outbreaks that had been predominantly localized in the countryside and rural villages and town, it soon invaded the capitals of Monrovia and Conakry, as well as other regional metropolitan centers.

The present situation has been qualified as a “catastrophic” epidemic “deteriorating daily” (*Médecins sans Frontières*, [Doctors without Borders], statement mid-August 2014) [1]. The October 2014 issue of the CDC journal Emerging Infectious Diseases (vol 20, No 10) provided a spotlight of our current over-arching knowledge on Ebola.

The current EVD trends are aggressive and fast spreading beyond the infection’s original regional boundaries. These characteristics, taken together with the extensive traveling that globalization presently entails, raise the specter of an emerging world-wide pandemic. WHO declared the current EBOV epidemic to be an international public health emergency on the 8th of August 2014.

On the 30th of September 2014, the first confirmed case of Ebola was diagnosed in the United States. By mid-September, cases had been confirmed in Nigeria, as well as Madrid, and suspected in France, Ireland, and Australia. Senegal, by aggressively shutting down its borders, declared itself a country “free of Ebola”. In a statement issued on the 26th of September of that same year, WHO deplored that “…*the Ebola epidemic ravaging parts of West Africa is the most severe acute public health emergency seen in modern times. Never before in recorded history has a biosafety level four pathogen infected so many people so quickly, over such a broad geographical area, for so long*…”, and announced that the number of diagnosed cases had surpassed 8,000, with a close to 50% fatality rate. Of particular concern was the fact that close to 250 healthcare workers were among the dead, partly due to the lack of, or misuse of the PPE equipment, and long exhaustive hours [1,2]. By the end of the month of October 2014, WHO statistics indicated over 13,703 known cases of Ebola worldwide, with 4,922 deaths (35.9% fatality rate).

With respect to PPE, the CDC recommended “…*including, at a minimum, disposable gloves, gown (fluid resistant/impermeable), eye protection (goggles or face shield), and facemask to protect against direct skin and mucous membrane exposure of cleaning chemicals, contamination, and splashes or spatters during environmental cleaning and disinfection activities. Additional barriers (e.g., leg covers, shoe covers) should be used as needed. If reusable heavy-duty gloves are used for cleaning and disinfecting, they should be disinfected and kept in the room or anteroom. Be sure that are instructed in the proper use of personal protective equipment including safe removal to prevent contaminating themselves or others in the process, and that contaminated equipment is disposed of appropriately*….” [2].

The CDC also instructed “…(to) *use a U.S. Environmental Protection Agency-registered hospital disinfectant with a label claim for a non- enveloped virus (e.g., norovirus, rotavirus, adenovirus, poliovirus) to disinfect environmental surfaces in rooms of patients with suspected or confirmed Ebola virus infection. Although there are no products with specific label claims against the Ebola virus, enveloped viruses such as Ebola are susceptible to a broad range of hospital disinfectants used to disinfect hard, non-porous surfaces. In contrast, non-enveloped viruses are more resistant to disinfectants. As a precaution, selection of a disinfectant product with a higher potency than what is normally required for an enveloped virus is being recommended at this time. EPA-registered hospital disinfectants with label claims against non-enveloped viruses (e.g., norovirus, rotavirus, adenovirus, poliovirus) are broadly antiviral and capable of inactivating both enveloped and non-enveloped viruses*…” [2]. The means of disinfection that are reported of common use in West Africa at the moment include copious drenching with bleach.

It has now come to light that the majority of health care workers worldwide do not have sufficient training in the PPE and disinfection protocols that EBOV contamination requires. Most hospitals in the US are reported not to have in stock the appropriate materials to ensure the safety of the nursing and medical staff. CDC and WHO issued updates and revisions of their protocols in mid October 2014. Taken together, this fluid situation contributes to the state of heightened alert, concern and anxiety among professionals and the general population. President Obama appointed Ron Klain as “Ebola Response Coordinator” to manage the national response efforts at diagnosis, quarantine, treatment and prevention of Ebola on the 17th of October 2014.

By the 6th of October 2014, the first case contracted on European soil was reported in Madrid, Spain: Teresa Romero, a doctor who had been caring for two missionary priests who had just died of Ebola in Madrid. This was the first case of Ebola diagnosed outside of the African continent. On the 8th of October 2014, Thomas Eric Duncan the first patient who developed Ebola on US soil, became the first Ebola patient to die on US soil. He had assisted a pregnant woman, apparently Ebola-negative (that is, showing no fever or other EVD symptoms as per the reports of her family and community healthcare personnel) at the time. She had collapsed due to excessive *pre-partum* bleeding on a street in Liberia on the 18th of September. On the 20th of September, Mr. Duncan had returned to the US. A few days later the woman gave birth to an apparently healthy baby, albeit premature by a few weeks. She continued to have extensive vaginal bleeding post-delivery and later died; the infant, deprived of her natural mother’s milk, was fed on artificial milk, but, whereas he had shown signs of stabilizing following the eventful birth, choked on the formula milk and died a few days later. According to official Liberian reports, neither mother nor infant had confirmed EVD; but, the popular press had drawn the association of Mr. Duncan falling ill with Ebola following his last good deed before coming to the US from Liberia, which was a long-planned trip to visit his son and his fiancée in Dallas. On the 24th of September, following his arrival in the US, Mr. Duncan developed the first symptoms of incipient fever. He sought hospitalization on the 26th of September, but was immediately discharged despite a temperature above 100°F, and having clearly stated to the hospital staff his recent arrival from Liberia. On the 28th of September, he returned to the hospital, was diagnosed and placed in isolation, and passed away from Ebola 10 days later. Mr. Duncan’s relatives and caregivers were placed in quarantine, but none of them developed fever or any EVD symptoms. Two of the nurses who cared for Mr. Duncan at the Texas hospital, Nina Pham and Amber Vinston, were the first two secondary cases of diagnosed Ebola outside Africa and on US soil. They both were successfully cured from the disease.

Assuming no change in the control measures for the present epidemic, the cumulative reported numbers predict an exponential growth of the epidemic, along with atrociously high numbers of Ebola-infected and Ebola fatalities by early 2015. In this, as in the previous Ebola outbreaks, the majority of Ebola patients are young adults 15 to 44 years of age (49.9% male), with an asymptomatic period of 1–21 days (median = 11.4 days; serial interval = 15.3 days). On the basis of the initial periods of exponential growth, the estimated basic reproduction numbers (R_0_) have been reported to be 1.71 (confidence interval, CI^95^: 1.44 to 2.01) for Guinea, 1.83 (CI^95^: 1.72 to 1.94) for Liberia, and 2.02 (CI^95^: 1.79 to 2.26) for Sierra Leone, the three *fulcrum* countries of this present outbreak. The estimated current reproduction numbers (R) are 1.81 (CI^95^: 1.60 to 2.03) for Guinea, 1.51 (CI^95^: 1.41 to 1.60) for Liberia, and 1.38 (CI^95^: 1.27 to 1.51) for Sierra Leone. Therefore, the corresponding doubling times were derived to be 15.7 days (CI^95^: 12.9 to 20.3) for Guinea, 23.6 days (CI^95^: 20.2 to 28.2) for Liberia, and 30.2 days (CI^95^: 23.6 to 42.3) for Sierra Leone [3]. These data suggest that different mutations of the Zaire species may characterize each of the three *fulcrum* countries. If molecular analyses should confirm this hypothesis, the difficulty and complexity to raise an effective vaccine would considerably increase. As things stand, CDC reports that, at the very best, an effective vaccine against EBOV could not be ready, tested and validated before early 2016.

## Review

### Ebola virus disease

#### The Ebola virus

The Ebola River flows through the hills and the planes of northern Zaire, now the Democratic Republic of the Congo. Ebola is the headstream of the Mongala River, a tributary of the Congo River. Along these banks, the Ebola virus was first discovered in the mid seventies. In 1998, the genus of that virus family was first introduced as the “Ebola-like viruses” (Vertebrate Virus Subcommittee proposal to the International Committee on Taxonomy of Viruses [ICTV]). By 2002 the international community came together to simplify the nomenclature, and changed its name to Ebola virus (EBOV, ebolavirus).

The genus ebolavirus, closely related to the Marburg virus, belongs to the negative strand RNA family of *Filoviridae*, order *Mononegavirales*. Negative-sense viral RNA is complementary to mRNA and thus must be converted to positive-sense RNA by an RNA polymerase before translation. As such, purified RNA of a negative-sense virus is not infectious by itself as it needs to be transcribed into positive-sense RNA, although each virion can be transcribed to several positive-sense infectious RNAs. RNA viruses in general, and negative sense RNA viruses in particular have high mutation rates compared to DNA viruses, because viral RNA polymerases lack the proof-reading ability of DNA polymerases. This property raises the difficulty in producing effective vaccines. It must also be noted that errors during reverse transcription are embedded into both strands of the intermediate viral DNA before integration into the host DNA, which can cause changes in the transcription-translation of the host cell functional household protein constituents, and consequential significant alterations in the host cell structure and function in its natural micro-environmental milieu. In the case of EBOV, the virus infects the myeloid population, and it is to be expected that EBOV-infected macrophages and dendritic cells may have structural and functional alterations that may impair their role in cell-mediated immunity and antigen presentation (*vide infra*).

The *Filoviridae* consist of three *genera*: cuevavirus, marburgvirus, and ebolavirus (EBOV). Five species of EBOV are now known, and four of these cause Ebola virus disease (EVD) in humans [4,5]. The five known species of EBOV are named for the region where each was originally identified:

- Bundibugyo virus: On the 24th of November 2007, the Uganda Ministry of Health confirmed an outbreak of Ebola in the Bundibugyo District, following confirmation of samples tested by the US National Reference Laboratories, CDC and WHO. On the 20th of February 2008, the Uganda Ministry officially announced the end of the epidemic in Bundibugyo, with the last infected person discharged on the 8th of January 2008. An epidemiological study conducted by WHO and Uganda Ministry of Health scientists determined there were 116 confirmed and probable cases, with a mortality rate of 34%.
- Reston virus: Reston EBOV was discovered during an outbreak of simian hemorrhagic fever virus in crab-eating macaques from Hazleton Laboratories (now Covance) in 1989. Since the initial outbreak in Reston, Virginia, it has since been found in nonhuman primates in Pennsylvania, Texas, and Siena, Italy. In all cases, the affected animals had been imported from a facility in the Philippines, where the virus has also infected pigs. Despite its status as a Level‑4 organism and its apparent pathogenicity in monkeys, Reston EBOV is not believed to cause EVD in exposed human laboratory workers. It is the only ebolavirus that is pathogenic for nonhuman primates, and that is nor originally endogenous to Africa.
- Sudan virus: Sudan EBOV emerged in 1976, and was at first assumed to be identical with the Zaire species. It is now thought that this species broke out first amongst cotton factory workers in Nzara, Sudan (today South Sudan). The first case reported was a worker exposed to a potential natural reservoir, although the original carrier remains unknown. The most recent outbreak occurred in August, 2014, when 13 cases were reported in Djera, Democratic Republic of Congo. The fatality rates for Sudan ebolavirus are variable, ranging from 54% in 1976, 68% in 1979, to 53% in 2000 and 2001.
- Taï Forest virus (i.e., Côte d’Ivoire ebolavirus): This species of EBOV was first discovered among chimpanzees from the Taï Forest in Côte d’Ivoire, Africa, in 1994. Necropsies showed blood within the heart to be brown; no obvious marks were seen on the organs; and one necropsy displayed lungs filled with blood. Taken together, this symptomatology is suggestive of tissue hypoxia secondary to methaemoglobinaemia, a blood disorder characterized by the presence of a higher than normal level of ferric [Fe3+] rather than ferrous [Fe2+] hemoglobin. Ferric hemoglobin has increased affinity for oxygen but reduced ability of the red blood cell to release oxygen to tissues, compared to the normal ferrous hemoglobin. Consequently, methemoglobin concentration is elevated, the blood appears oxidized and acquires a deep red-to-brown color, and tissue hypoxia occurs. At elevated concentrations, methemoglobin can cause coma and death; at intermediate concentrations (about 50%), it contributes to headaches, confusion, agitation and related behavior problems; even at lower concentrations (below 30%), methemoglobin can contribute to severe alterations and histopathologic dysplasias of mucous membranes or the oral and the gastro-intestinal lining. Hemoglobin is also found in non-blood tissues and cells, such as neurons and macrophages, where its function is primarily that of an anti-oxidant. At high concentrations of methemoglobin, that anti-oxidant protective ability is lost, placing both neurons and myeloid cells at higher risk of programmed cell death (i.e., apoptosis). Since hemoglobin synthesis begins during the pro-erythroblast stage of the red blood cell cycle, EBOV-mediated disruption of the process can lead to the production of ferric (Fe3+), rather than ferrous (Fe2+) hemoglobin. The synthesis of the heme component takes place in the mitochondria with the condensation of glycine & succinyl-CoA to form δ-aminolevulinic acid, which then leaves the mitochondria and forms porphobilinogen, and eventually coproporphyrinogen. In that form, it re-enters the mitochondria and becomes protoporphyrin. Protoporphyrin combines with iron to form the heme, which now exits the mitochondria to combine with the globin molecule produced in the ribosome. Studies of tissues taken from the chimpanzees showed results similar to human cases during the 1976 Ebola outbreaks in Zaire and Sudan. As more dead chimpanzees were discovered, many tested positive for Ebola using molecular techniques. The source of the virus was traced to infected western red colobus monkeys (*Procolobus badius*) upon which the chimpanzees preyed. One of the scientists performing the necropsies on the infected chimpanzees contracted EBOV, developed symptoms similar to those of dengue fever within 7 days, was treated aggressively and fully recovered within 6 weeks.
- Zaire virus: Zaire EBOV is the type reference for all species of Ebola virus, since it has a sole known member - although it is suspected to have the potential for several mutant forms. Zaire EBOV has the highest mortality rate of the Ebola virus, with a case-fatality rate up to 90% in some epidemics, and an average case-fatality rate of approximately 83% over 27 years. Zaire EBOV is responsible for the largest number of outbreaks of the five known members of the *genus*, including both the first documented outbreak in 1976, and the current most deadly outbreak. Originally, the symptoms were mistaken for malaria, and the patients received quinine, with unsuccessful outcomes.

As noted, all EBOV’s are enveloped non-segmented negative strand RNA viruses characterized [4-6] by:

- A lipid bilayer coat that protects the viral genome and facilitates host cell entry
- A viral genome with several gene overlaps
- A fourth gene that encodes four proteins that include
  - ○ soluble glycoprotein, sGP;
  - ○ small soluble glycoprotein, ssGP;
  - ○ Δ-peptide, and
  - ○ viral glycoprotein heterodimer 1,2 (vGP_1,2_) using co-transcriptional editing to express ssGP and GP1,2, and proteolytic cleavage to express sGP and Δ-peptide
- A peak of infectivity of its virion that is associated with particles ≈ 805 nm in length
- A genome that differs from that of its related family member, the Marburgvirus (MBGV), by ≥50%, but that diverges from the genome of other EBOV by <50% at the nucleotide level, and
- A set of virions that shows almost no antigenic cross reactivity with Marburg virions.

The natural reservoir host of Ebola virus remains unknown. However, on the basis of evidence and the nature of similar viruses, EBOV is believed to be animal-borne and that bats are the most likely reservoir. Four of the five virus strains occur in an animal host native to Africa. Fruit bats of the *Pteropodidae* family are the most likely natural ebolavirus hosts. Bats and other wild animals drop partially eaten fruits and pulp, and land mammals may feed on these fallen fruits, thus initiating the EBOV-infected food chain that leads to human consumption.

In brief, it is believed that Ebola is introduced into the human population through close contact with the blood, secretions, organs and other bodily fluids of infected animals such as the fruit bats, chimpanzees, gorillas and other monkeys, forest antelope and porcupines, which all have been found ill or dead with signs akin to EVD in the rainforest. Fruit production, animal behavior, as well as other indigenous factors of sub-tropical Africa that vary from year to year and in different seasons and places may contribute to trigger outbreaks of the different EBOV species among animal populations and humans, although no clear corollary relationships have been uncovered to be predictive of Ebola outbreaks [1,2].

Whereas EBOV is acquired upon contact with blood or bodily fluids of infected animals, it is then thought to spread through human-to-human transmission only via direct contact (through broken skin or mucous membranes) with blood, secretions, or other bodily fluids of infected people, and possibly with surfaces and materials (e.g. bedding, clothing) contaminated with these fluids. In addition, traditional caregiving, which may engage physical contact and touching, and burial ceremonies during which mourners have direct contact with the body of the deceased person are thought to play an important role in the transmission of Ebola (*vide infra*) [1].

At this time, and based on the epidemiological patterns of previous outbreaks, it is not believed that the virus can be contracted by aerosol, although the specter of mutated EBOV that could be airborne is a distinct possibility, if not a statistical probability. The aggressive nature of the outbreak presently in course seems to suggest that the current species of EBOV may in fact already have mutated and evolved into a more virulent form than originally expected, with contagion potential beyond exposure to body fluids of patients actively showing symptoms, as Richard E. Besser, MD, former CDC acting director, suggests. It is possible, he and others at CDC argue, that the Ebola virus could already spread “…*through the air in tight quarters*…”; and Tim Skinner, CDC spokesman, added that CDC is presently exploring whether and how the US should modify its policy to face this world-wide health threat. The contributions of environmental factors to EBOV transmission are not fully understood to this date. It is, therefore, most appropriate for CDC to emphasize the importance of avoiding “…*contamination of reusable porous surfaces that cannot be made single use. Use only a mattress and pillow with plastic or other covering that fluids* (sic*) cannot get through. Do not place patients with suspected or confirmed Ebola virus infection in carpeted rooms and remove all upholstered furniture and decorative curtains from patient rooms before use. To reduce exposure among staff to potentially contaminated textiles (cloth products) while laundering, discard all linens, non-fluid-impermeable pillows or mattresses, and textile privacy curtains into the waste stream and disposed of appropriately*…” [1,2].

Moreover, it is widely believed that transmission of EBOV can only occur from a patient who clearly manifests symptoms [1,2]; however, “…*there is no proof that a person infected – but lacking symptoms – could not spread the virus to others*…” (David Willman, Ebola experts urge greater caution, Los Angeles Times, Tuesday October 7, pp. A1 & A10). In fact, the rationale for this position, strictly on immunopathological grounds is unclear at best and at worst categorically unfounded. The statement is apparently based on the findings obtained from the previous outbreaks. Nonetheless, scant data are available to generate statistical inference of sufficient power to warrant such assertion. In brief, it is possible, or at least not improbable, based on biological and statistical evaluation of the evidence, that EBOV is contagious well before symptoms are noted. This, in and of itself, may contribute to explain why the current outbreak is aggressively advancing beyond our comprehension and expectation. Indeed, EBOV+ survivors are infectious as long as their blood and body fluids, including semen and breast milk, contain the virus [1], whether or not they manifest obvious and evident symptoms. Male survivors seem to be able to transmit the disease via semen for nearly two-to-three months following the date they are declared “cured”.

Based on the observations gathered during the previous outbreaks, it is believed that EBOV is sensitive to heat and chemical denaturation. Common expectations are that it can be degraded by heat (30–60 minutes at 60°C or boiling for 5 minutes), or by some lipid solvents (e.g., alcohol-based products, detergents, sodium or calcium hypochlorite). But supportive evidence across the recognized EBOV species is not compelling to this date. Evidence suggests, by contrast, that EBOV may have remarkable resistance. The virus is suspected to survive on hard and porous surfaces soiled with body fluids of EBOV+ patients, such as bedding, clothes, walls and other planes, for several days. To cleanse EBOV-contaminated requires extensive washing with chlorine and ethanol diluted in clean uncontaminated water (i.e., one part chlorine, one part ethanol, nine parts clean - possibly distilled or double-distilled -water). It is in part for these reasons, that the dearth of clean water remains a critical impediment in slowing the outbreak and bringing about an effective medical response in West Africa [6,7].

Quarantine, which is nothing but enforced isolation, is currently the most effective mode of decreasing the spread of this disease. Households, schools, localities, villages, town, cities or entire regions may be lawfully isolated where the disease is occurring or believed to occur, or individuals putatively infected may frequent. In this fashion, Senegal recently declared itself a “Ebola-free” country. Contact tracing is an important approach, perhaps one of the most effective public health protocols to contain an outbreak. Contact tracing permits to find everyone who has had close contact with infected individuals and watching for signs of illness. If any of these contacts come down with the disease, they should be isolated, tested, and treated, and their contacts traced and followed. The process is systematically repeated by tracing the contacts’ contacts. It is clear that contact tracing is feasible and efficient only when the number of cases is small or limited; as an epidemic grows, contact tracing evidently takes an increasingly prohibitive amount of time and manpower to run.

### From epidemic outbreaks to pandemic threat

#### Ebola pathology

Ebola virus disease (EVD), Ebola hemorrhagic fever (EHF), or simply Ebola is a disease of humans and other primates caused by EBOV. Symptoms of Ebola can start 2–21 days following initial contact with the virus. Initial manifestations include a sharp rise in fever, sore throat, muscle pain and headaches. Explosive vomiting and diarrhea soon follow, which, if left untreated, lead to serious dehydration, dizziness, confusion, kidney and liver failure, and eventually complete physiological collapse. Skin rash and eruptions appear, which soon develop into bleeding infected ulcerations. Extensive internal and external bleeding from the orifices (i.e., eyes, nose, ears; bloody diarrhea and urine), and signs of internal hemorrhage, including alterations in the integrity of the blood–brain barrier (BBB) (Brakhordarian et al., in press), contribute to the profuse and cascading loss of micronutrients. Laboratory findings confirm low white blood cell and platelet counts, and elevated liver enzymes.

The general symptomatic profile of EVD resembles that of other viral hemorrhagic fever (VHF) diseases [6,8-10], including dengue (Chiappelli et al., Bioinformation, in press). Consequently, the diagnosis for EVD can be complicated and relatively slow, because several of its symptoms overlap with malaria, cholera, dengue, and other VHFs. Expectations are, in fact, that, as the present Ebola epidemic continues to grow, it may eventually shut down malaria and dengue control efforts on the African continent. Case in point, the present outbreak has virtually hampered malaria control efforts in Liberia, Guinea and Sierra Leone, raising fears that cases of the mosquito-borne illness will soon rise because routine health care in the affected countries has all but collapsed (Thomas Teuscher, acting executive director of *Roll Back Malaria Partnership*). As a result, and in part because the early symptoms of Ebola mimic the symptoms of malaria, tens of thousands of people are erroneously be given antimalarial medication, which can induce drug resistance to the parasite that causes the disease in malaria-free individuals, and deplete the available doses of anti-malaria medicine. Misdiagnosis and mistreatment may thus not only delay prompt intervention in EBOV+ patients, but also contribute to the resurgence of malaria in Africa and globally. Indeed, people who are free of Ebola are at higher risk for malaria in West Africa presently, as noted by Estrella Lasry, MD, tropical medicine specialist for *Médecins sans Frontières.*

Patients with severe hemorrhagic disease, such as Ebola or dengue produce dangerously high levels of inflammatory cytokines, which destroy normal tissue and microcirculation, leading to profound capillary leakage, renal failure, and disseminated intravascular coagulation. For the discriminant diagnosis of Ebola, malaria and other hemorrhagic diseases must first be excluded, and the diagnosis of EVD must then be confirmed by analysis of blood samples, and possibly other body fluids including saliva, for EBOV antibodies, EBOV RNA, or EBOV itself [1]. Taqman-RT-PCR protocols for the detection of the various species of EBOV, including Zaire-EBOV, were developed and established on a portable Smartcycler-TD over a decade ago [11,12]. However, the use of this technology is often not feasible on the ground and in field hospitals of isolated villages or localities of West Africa. Confirmation of EBOV infection can also be obtained by electron microscopy, and virus isolation by cell culture [1]. To confirm Ebola cases, samples must be shipped to a testing site; however, considering the high infectivity of these samples, shipping operations such as these are of very high risk.

#### Proteomic signature

Genomic and proteomic advances now proffer some degree of understanding of the EBOV virion. Data propose a minimal gene set that can distinguish between EVD survivor and non-survivor non-human primate experimental animals under specific anticoagulant treatment conditions. In a recent study of 23 rhesus macaques: 19 treated with anticoagulant therapeutics (11 with recombinant human activated protein C, and eight with recombinant nematode anticoagulant protein – of these animals, four of the treated, two in each group, survived a lethal challenge with EBOV [H.sapiens-tc/COD/1995/Kikwit-9510621]) and four untreated controls, the findings of transcripts micro-analyses revealed that there might be few genes that could be identified as prognostic biomarkers of survival [13]. Analysis of mRNA expression changes between treated EVD survivors and treated/untreated non-survivors further revealed 20 unique identified probes (16 annotated genes, three genetic loci, one microRNA) that distinguish EBOV-challenge survivors from non-survivors. By means of the statistical technique of hierarchical clustering and leave-one-out cross-validation, the minimal gene set could be correctly classified with nearly 100% accuracy [13]. Specifically, the data pointed to:

- The up-regulation of six genes
  - ○ ACCN1, Amiloride-sensitive cation channel neuron 1, a member of the degenerin/epithelial sodium channel superfamily that contributes to the regulation of neurotransmission.
  - ○ CEBPE, CCAAT/enhancer binding protein-ε, a pro-apoptotic gene product that is essential for terminal differentiation and functional maturation of committed granulocyte progenitor cells (a defect, or decrease in this gene product resulting in granules deficiency, and impaired functional myeloid differentiation and maturation [14].
  - ○ CRHR2, Corticotropin releasing hormone receptor 2
  - ○ FAM63A, putative cytoskeletal protein
  - ○ HMP19, Neuron-specific protein family member 2
  - ○ IL2RA, Interleukin-2 receptor-α, CD25
  - ○ LTF, lactoferrin of the transferrin family, a 80 kDa glycoprotein widely represented in body fluids (i.e., milk, serum, saliva, tears, nasal secretions), as well as secondary granules of granulocytes and other myeloid cells, and as such an essential component of cellular immunity. Whereas administration of lactoferrin has been shown to be beneficial in reestablishing physiological homeostasis of iron metabolism in an experimental model of hemorrhagic disease in rodents [15], the putative palliative role of lactoferrin administration in hemorrhagic EVD remains to be examined.
  - ○ PSMA1, Proteasome subunit alpha type-1, a multi-catalytic proteinase complex in proteasomes, which cleaves peptides in an ATP/ubiquitin-dependent process in a non-lysosomal pathway.
  - ○ RCHY1, RING finger and CHY zinc finger domain-containing protein 1 that has ubiquitin-protein ligase activity, and binds with p53 and promotes the ubiquitin-mediated proteosomal degradation of p53, thus being oncogenic in nature.
  - ○ SLC9A7, solute carrier family 9, subfamily A, member 7, a Na/K proton antiporter, member of the solute carrier family 9 protein family, that is primarily localized to the trans-Golgi network and is involved in maintaining pH homeostasis in organelles along the secretory and endocytic pathways.
- The down-regulation of 10 genes
- The following genetic loci
  - ○ AC009283: chromosome 17
  - ○ LOC100289371: mitochondria-encoded cytochrome c oxidase III pseudogene, chromosome 5
  - ○ LOC440871: chromosome 2
- microRNA (miR-122), a known positive regulator of viral replication for hepatitis C virus [16] and in fact a biomarker of neuroinflammation in patients with chronic hepatitis C virus [17] – indeed, preliminary trials show that miravirsen, the Santaris Pharma locked nucleic acid-based antisense oligonucleotide that can delivered to the liver to effectively inhibit miR-122 following intravenous injection without liver toxicity, significantly reduces reduced hepatitis C virus viremia in non-human primates (i.e., chimpanzees) [18]. Testing of miravirsen, as an experimental RNA therapy, in EBOV-infected patients, albeit seemingly promising [19], has not commenced as of this date.

In the transcriptomic analysis of EBOV-challenged non-human primates study noted above, overall, survivors exhibited “*significant and opposing regulation of expression*” when compared to non-survivors, without regard to whether the non-survivors received anticoagulant or not. Specific analysis of the identified genes suggested that genes associated with cellular immune and inflammatory responses were up-regulated in non-survivors, consistent with the hypothesis that non-survivors experience acute deregulation of cellular immune surveillance [13] (*vide infra*). It is possible and even probable that future investigation of these pathways could reveal transcriptional signatures specific to EBOV infection and host immune response.

Rather than the more invasive technique of collecting blood for measuring EBOV, saliva was established in earlier studies to be potentially useful and promising for the investigation of Ebola epidemics and pandemics. In those studies, anti-EBOV immunoglobulin G antibodies could not be detected in the saliva of patients who had elevated levels of serum anti-EBOV immunoglobulin G antibodies by enzyme-linked immunosorbent assay. By contrast, both salivary and serum samples from these patients exhibited positive serum RT-PCR for EBOV [12]. Transcriptomic analysis of the type described above must now be performed on salivary samples, in order to maximize utilization of this body fluid for the purpose of fast, sensitive and reliable diagnosis EBOV infection.

For the time being, no specific treatment for EVD disease is available, in part because of the complex, multi-step and highly orchestrated path of internalization and fusion of the Ebola virus. Cellular entry of EBOV requires a series of cellular protein interactions and molecular mechanisms, which commence with the interaction of the heavily glycosylated viral glycoprotein, GP_1,2_, with both adherence factors, such as the Δ-peptides and receptors on the surface of host cells, such as the T cell immunoglobulin and mucin domain 1 (TIM-1) on epithelial cells. Upon receptor binding, the virus is internalized into endosomes primarily via macro-pinocytosis. The acidified endosomal cellular proteases Cathepsin L and B cleave GP_1,2_, and expose residues in the receptor binding site. These viral residues interact with endosomal/lysosomal membranes that contain the Niemann Pick C1 like protein [19]. Indeed, Niemann Pick C1 protein expression is required for productive EBOV infection. That the drug ezetimibe blocks Niemann Pick C1 like protein [20] seems to suggest that it might well contribute to blunt productive EBOV infection. However, this treatment option has not been examined in EVD patients at this time. Certain novel drugs are undergoing testing, such as the human immunodeficiency virus-inactivating protein cyanovirin-N, which binds to vGP1,2, and inhibits infectivity of Ebola virus [21,22].

#### Medical anthropology

There are certain requirements that must be met when attempting to prevent EVD epidemics to burgeon into a pandemic threat, and which can be summarized as follows:

1. correct the deficiency in the development and implementation of surveillance response systems against Ebola specifically, and others infectious disease outbreaks in Africa more generally, by means of a well coordinated program of evidence dissemination such as that we proposed in translational science for evidence-based health care dissemination [23-25],
2. correct the lack of education and knowledge resulting in an EVD outbreak, which contributes to trigger panic, anxiety, psychosocial trauma, isolation and dignity impounding, stigmatization, community ostracism and resistance to associated socio-ecological and public health consequences, by utilizing dissemination efforts toward increasing health literacy [25,26];
3. correct the limitations in financial resources, human technical capacity and weak community and national health system operational plans for prevention and control responses, practices and management;
4. correct the inadequate leadership and coordination; and
5. correct the lack of development of new strategies, tools and approaches, such as improved diagnostics and novel therapies including vaccines, which can assist in preventing, controlling and containing Ebola, by emphasizing evidence-based, patient-centered and effectiveness-focused concerted interventions [27].

As noted above, Ebola is caused by one of five identified EBOV viruses, and is highly contagious. Human-to-human transmission can occur via direct contact with blood or bodily fluids from an infected person, including touching during caregiving, embalming of an infected dead person, contact with the corpse during burial, or handling personal objects of the Ebola victim contaminated by the virus, particularly bedding, clothing, needles and syringes [28].

#### Epidemiologic transitions and zoonotic threats

The “third epidemiologic transition” is characterized by an unprecedented number of novel diseases detected over the last four decades, with “*an increased incidence and prevalence of pre-existing infectious diseases that were thought to be under better control*” ([29], p. 256). Many emerging pathogens are generating antibiotic resistance, and some are multi-drug resistant. All of this occurs “*within the broader context of globalization, involving international trade, migration, information networks, and the convergence of human disease ecologies*” ([29], p. 257). Disease pathogens, as with Ebola, “*are often provided the opportunity to jump the ‘species barrier’ by a combination of ecological disruption and increased contact between humans and wild reservoir species. The size and mobility of human populations increases the potential for the pathogen to escape its geographic barrier*” ([29], p. 259), and is complicated by the widening of inequalities within and between societies increases the spread of emerging and reemerging diseases [30]. Most pathogens in humans are zoonotic in origin. Population, ecological, and behavioral alterations that increase contact with wildlife intensify the emergence of these pathogens. Anthropogenic environmental modification has altered the risk of zoonotic infection from wildlife. Ebola exemplifies how anthropogenic factors facilitate pathogen transmission between human and nonhuman animal populations [31]. Emerging infectious diseases (EIDs) of free-living wild animals are “*associated with ‘spill-over’ from domestic animals to wildlife populations living in proximity; and human intervention via host or parasite translocations*” ([32], p. 443). The increasing populations of domestic animals, especially in feedlots, with close and constant contact with wildlife contribute to transmission. As a result, many wildlife species are reservoirs of pathogens that threaten the health of domestic animals and humans. Disease emergence most frequently results from a change of ecology of host and pathogen, or both. Human population expansion has driven the emergence of EIDs through increasing population density, especially in urban areas, and encroachment in to wildlife habitat. Human encroachment on shrinking wildlife habitat has also caused increased wildlife population densities and the emergence of wildlife EIDs. Wild animals play an especially important role in EID emergence by “*providing a ‘zoonotic pool’ from which new pathogens can emerge*” ([32], p. 446). Understanding how environmental change alters the contact between human and non-human primates, thereby increasing the possibility of sharing infectious diseases detrimental to humans or nonhuman primates, is essential to global health planning. Diseases like Ebola devastate both human and wildlife communities, with outbreaks of the virus tracing their origins to zoonotic transmissions from local apes, especially since monkeys and apes often share parasites with humans [33]. However, human displacements of fruit bats through deforestation make this a vector species for transmission of Ebola, as well.

#### Human-animal entanglements

Human-animal “entanglement” focuses on the ways in which disease, instead of alienating humans from other life forms, brings their intimate relationships into sharper relief [34]. As such, people, animals, “*pathogens and spaces are connected in a process of ‘mutual becoming’*” ([34], p. 69). Health thus becomes the locus for critically analyzing “the production of life, where life is the ongoing, dynamic result of human and nonhuman interactions over time” ([34], p. 60). Studies of EIDs, such as Ebola, bring human and nonhuman animals together within a framework that examines “*how anthropogenic landscape change leads to ‘disease emergence’*” ([34], p. 69). An example of such entanglement is bush meat hunting, which has brought about “*unsustainable and increasing rates of harvesting, leading to the ‘empty forests’ of parts of Africa*” ([35], p. 366). EIDs, such as Ebola, which have the highest fatality rates, have emerged from wildlife. Moreover, the causes of their emergence are clearly linked to the way in which humans interact with wildlife, through encroachment, road building, deforestation, hunting and the global trade in them. The recent interest in the underlying causes of zoonotic disease emergence, namely changes to human demography, behavior and the environment, denotes a shift in tropical medicine, from reactive to proactive approaches. An anthropology of viral hemorrhagic fevers (VHFs) focuses on “the hotspot” to understand the “*two routes of VHF infection: from animal reservoirs to humans and between humans*” ([36], p. 280). Through this perspective, human–animal entanglements are viewed in terms of the social production of space to understand viral movement by elaborating the circumstances through which viruses, humans, objects, and animals come into contact. The “*material proximities between animals, humans, and objects that constitute the hotspot*,” ([36], p. 280) can establish an epistemological “frontier” for collaborative efforts between anthropologists and other scientists on behalf of VHF management and control.

#### Ebola through an anthropological lens

Anthropological studies in these hotspots aim at understanding local views and responses to an outbreak. In experiencing Ebola, local people employ multiple explanatory models to make sense of and respond to the outbreak; their indigenous epidemic control measures are often implemented and these are consistent with the ones being promoted by healthcare workers; although some cultural practices, such as burial practices, amplify the outbreak [37]. How local people in Gabon viewed Ebola and responded to the rapid killer during outbreaks in the 1990s, and how they view the international health care worker teams mobilized to help control the disease are key concerns in understanding subsequent outbreaks [38]. The fear of being infected with Ebola and the desire to flee the area are human universals. Local people in village communities invoked sorcery and consulted indigenous healers as they attempted to understand and explain the disease. At first, they questioned why particular individuals were infected with Ebola, and later as the epidemic progressed, why the virus emerged in their country, noting the impact of European imperial encroachment on and “poisoning” of the forest. Local people also worked to help control the outbreak by isolating infected people or limiting their contacts, kept children away from the sick, and sought treatment from indigenous healers and government health care workers. Localities in Gabon also felt exploited by international health workers who passed through villages to draw blood, and who not only failed to compensate or report findings back to village communities, but also showed scant compassion or empathy for villagers’ expressions of loss of their loved ones.

#### The primacy of caring

Efforts to control high mortality diseases, such as Ebola, provide two distinct but complementary interventions during the first phases of an outbreak in the Republic of Congo [39]. The first approach emphasized understanding local people’s cultural models and political-economic explanations for the disease while the second approach focused on providing more humanitarian care of patients by identifying and incorporating local beliefs and practices into patient care and response efforts. The experiences of health care workers during Ebola outbreaks in Uganda in 2000–2001, Republic of the Congo in 2003, and Democratic Republic of the Congo in 1995 included: a lack of protective gear, basic equipment, and other resources necessary to provide care, especially during the early phases of the outbreaks; and stigmatization by family, coworkers, and community [40]. Improving treatment of VHFs in sub-Saharan Africa ultimately hinges on effective and compassionate care for the effected patient. To this end, there is the need to refocus efforts on aggressive supportive care and clinical monitoring; including communications and social mobilization experts as a primary part of every outbreak response team; and reestablishing the isolation ward as the key functional component of the overall outbreak control strategy [41]. The response efforts of *Médecins sans Frontières*, together with WHO, and the government health ministry to contain a VHF epidemic in Angola offer insight into care for those infected included “*community epidemiological surveillance, clinical assessment and isolation of patients, safe burials and disinfection, home-based risk reduction, peripheral health facility support, psychosocial support, and information and education campaigns*” ([42], p. 162). The key lesson learned is that containment of the Angolan epidemic depended on the collaboration of the affected community; a critical factor being active involvement of all stakeholders at the start of the outbreak response. In an attempt to understand social responses to a major Ebola outbreak in Uganda in 2000–2001, in local, national, and international contexts, over the course of the outbreak, a content analysis was conducted using articles, editorials, cartoons, and letters to the editor from the country’s two main English language newspapers [43]. Key themes were:

- social psychological responses, such as confusion, anger, and serious stigma;
- overworked medical personnel, accompanied by burnout;
- patients fleeing from hospitals;
- invocation of spiritual forces;
- varieties of national control strategies; and
- precautionary measures, such as international travel restrictions.

VHF outbreaks in Africa can be seen as a paradigm for ethical issues posed by epidemic emergencies, including isolation and quarantine; privacy and confidentiality; and the interpretation of ethical norms across different ethnocultural settings, especially research ethics [44]. While informed consent and institutional review processes governing collection of biological samples and epidemiological data are regulated by national guidelines and international conventions, there remains a need for new ethical frameworks on behalf of engaging potentially affected communities in research design and in the community permission process during an outbreak.

#### Framing Ebola: competing perspectives & the politics of knowledge

Four “narratives” about VHF frame the debates surrounding “cultural models” of risk and vulnerability; the intersection of social, ecological and disease processes; and the discourse on local knowledge [45]:

- the first concerns VHFs as an emerging global problem;
- the second views the problem as a deadly local disease requiring rapid mobilization and containment;
- the third understands local knowledge and socio-cultural practices as necessary to informing explanatory models of VHFs; and
- the fourth addresses longer term social and environmental interactions involved with disease patterns and vulnerabilities.

Together, they help shape “contrasting institutional and policy pathways” for responding to VHFs. Hence, zoonotic diseases afford an opportunity to pose scientific and policy challenges optimally met by complex modeling approaches of epidemiological, ecological and social processes. Moreover, the models, themselves, have “social and political lives,” because of the norms and values embedded in their design and intent [46]. Ebola, especially because of the fear of widespread contagion and death, provides a way to understand how the social and political lives of a zoonotic disease model can be co-constructed, scientifically and politically, with particular policy interests in mind.

#### Biosocial approaches

Outbreaks of Ebola remind us of the need for models of disease emergence that are “dynamic, systemic, and critical”; models that “incorporate change and complexity, and are global yet alive to local variation,” account for social inequalities, and then question “*how large-scale social forces influence unequally positioned individuals in increasingly interconnected populations*” ([47], p. 259). Regarding the interplay of culture and ecology in the epidemiology of infectious disease, behaviors related to diet, activity patterns, water use, and sexual practices that predispose individuals to certain diseases might be shaped by culture ([48], p. 254). While cultural practices, including agriculture and warfare, provide the means for people to actively transform their environments, these can also affect their susceptibility to infections. Cultural models of etiology, patient role and treatment influence how people respond to the illness ([48], p. 254). Human biosocial factors clearly have an impact on R0, the basic reproductive rate of a pathogen, or the expected number of infections caused by one infected individual in a susceptible population. “*All three components of R0 (contact or exposure rates, probability of transmission, and duration of infection) are partly regulated socially*” ([49], p. 1885). Social relationship patterns drive contact rates, and are themselves related to norms of social affiliation. Social disparities affect “*susceptibility to, and severity and duration of, infections as a result of psychosocial stress and risk for co-infection*” ([49], p. 1885). Social processes are central to causal complexes, including agricultural development and changing land uses, which increase vector-borne disease risk; together with “*the emergence of viral zoonoses; and social and economic activities, such as gathering of fruits, handling of bush meat, and rearing of animals, which bring people closer to natural or amplifying hosts, or their tissues*” ([49], p. 1885).

#### Critical medical anthropology

The term “syndemic,” is used to label the synergistic interaction of two or more coexistent diseases and resultant excess burden of disease within the context of structural factors [50]. A syndemical research framework attempts to explain co-infection and synergistic interaction of diseases and social conditions at the biological and population levels, especially with infectious diseases, “*their entwinement with each other, and with the social conditions and bio-psychological consequences of disparity, discrimination, and structural violence*” ([50], p. 423). The syndemic concept has been expanded to include how animal health and human health connect as “one health,” especially in prevention, where animal and human diseases, together with environmental degradation are influenced by social conditions and political economic arrangement [51]. While infectious disease epidemics result from long-term and complex social, ecological, economic and political processes, outbreaks are experienced “on the ground” as unexpected eruptions [52]. The dialectical relationship between the outbreak as an event and a process provides an opportunity to analyze epidemic temporality in the context of emergent infectious disease discourse and intensifying bio-political surveillance aimed at averting the “next pandemic.” This dialectic between an event and a process resides at the interface of states and markets as seen in the borderland of Uganda and the Democratic Republic of the Congo after an Ebola outbreak in 2008 [53]. Border conditions, in this case, underscore the centrality of the reconstructed “post-conflict state” as it attempts to regulate political space and restore regional economic development in the aftermath of an outbreak and regional civil warfare. Efforts to regulate national border traffic and commerce invariably met with entrenched forms of formal and “informal” economies, and often illegal trade in minerals and bio-resources by ordinary people engaged in cross-border commerce, many of whom viewed the reconfigured state as predatory. Cross-border trade and economies were disrupted, first by disease and warfare, and then by what was perceived, on the ground, as an attempt by “the predatory state” to regulate the everyday lives of localities and their citizens, who despite these disruptions, prevailed in reconstituting local markets based upon traditional livelihood practices. Only by considering both the activities of the state and the behaviors of the pathogen, in other words bringing together the macro- and micro-levels of analysis, can we understand an infectious disease like Ebola.

#### Biosecurity

The intersection of national security and public health in recent decades has brought about a reframing of the threat of infectious disease, from prevention to preparedness [54]. The norm of preparedness, used throughout the post-war decades in military and civil defense contexts, has migrated to governmental public health intervention. With this move, widespread public discussion of biological threats has focused on expert techniques, such as the scenario-based exercise, which serve to generate both a sense of urgency, absent the event itself, and knowledge about vulnerabilities in response capability, on behalf of intervention design. With the shift from population-based risk calculations to a focus on preparedness through scenario-based projections, “health” and “security” now shape biosecurity debates, including emergency management [55]. However, a key focus of the biosecurity discourse, that of “communication,” has not been fully conceptualized. “Bio-communicability” is a term used to indicate the cultural modeling of how this discourse is produced, circulates, and is received. Biosecurity is also an “object in the making” ([56], p. 1) under post-Cold War globalization, as it informs questions of global health, capitalism, neoliberalism, humanitarianism, citizenship, science, medicine, technology, ecology, surveillance, and risk. Recent ethnographies detail the ways “*actors and institutions in the Global North and the Global South”* ([56], p. 1) view both biological risks and the spread of dangerous biological entities that threaten individual and population health. Hence, a new “landscape” of global health policy and practices has transformed infectious disease prevention and control. This reconfiguration of “*institutions, technologies and financial flows*” ([57], p.199) includes framing of health as a security issue; the emergence of new global regimes and organizational assemblages focusing on specific infectious threats; the growth of humanitarian medicine; investments by global philanthropy in development assistance for health, especially in Africa, and the global trade in pharmaceuticals. These trends impact health ministries, as they interact with bilateral and non-governmental aid organizations, often through public-private partnerships. The resulting mandated managerial mechanisms to track funds, together with monitoring and evaluation protocols, are indicative of broad neoliberal reforms in the global health sector.

### Ebola pathology

#### Molecular pathology

Ebola hemorrhagic fever is a severe viral infection with fulminating pathology characterized by fever, shock and coagulation defects. Endothelial cells, macrophages, monocytes, and liver cells are the main targets of infection.

Following infection by the virus, a secreted glycoprotein (sGP) known as the Ebola virus glycoprotein (GP) is synthesized. Ebola replication overwhelms protein synthesis of infected cells and host immune defenses. The GP forms a trimeric complex, which binds the virus to the endothelial cells lining the interior surface of blood vessels. The sGP forms a dimeric protein that interferes with the signaling of neutrophils, allowing the virus to evade the immune system by inhibiting early steps of neutrophil activation. Neutrophils also serve as carriers to transport the virus throughout the entire body to places such as the lymph nodes, liver, lungs, and spleen [57].

EBOV infects dendritic cells, which in turn disables the interferon system and disrupts henceforth the host antiviral immune surveillance response [58,59]. Almost contemporaneously, the virus targets myeloid cells (i.e., monocytes/macrophages) and induces alterations in the blood clotting pathway, which together lead to significant increase in pro-inflammatory cytokines, including IL6, IL1-β and TNF-α, as well as nitric oxide, which damages the lining of blood vessels [60]. The virus operates in a sequential fashion by first disabling the immune system, then the vascular system, during which observed symptoms can include hemorrhage, hypotension, drop in blood pressure, and catastrophic organ failure. These symptoms are usually followed by shock and death.

After infection, IL10 levels were significantly elevated at the earliest times of infection in pediatric patients who died. The role of IL10 in inhibiting antigen-stimulated T cell proliferation supports the assumption that a T cell–mediated response is critical for survival during EVD. Levels of soluble intracellular adhesion molecule (sICAM) 1, and soluble vascular cell adhesion molecule (sVCAM) 1 for children are normally higher than for adults, and the levels that reported in surviving pediatric patients were consistent with these normal levels. Pediatric patients who died had sICAM and sVCAM levels 2–3 times above the reference range 0–10 days after symptom onset, but these levels dropped to within the reference range at 11–15 days. This pattern may reflect early excessive, and ultimately detrimental, endothelial activation in these patients. Consistent with this hypothesis are the increased plasminogen activator inhibitor 1 (PAI-1) levels also seen in pediatric patients who died; endothelial cells in response to activating cytokines release PAI-1. An overactive endothelial response, as evidenced by elevated sICAM, sVCAM, and PAI-1 levels, may be associated with death in children and adolescents. However, adult patients typically do not seem to be affected by this phenomenon. Elevated PAI-1 levels in pediatric patients with hemorrhagic manifestations likely represent the overactive endothelium and not a functional inhibition of fibrinolysis, since PAI-1 activity is likely to be low, as it rapidly converts to the inactive form under physiologic conditions. In brief, Ebola-surviving pediatric patients tend to have higher levels of the chemokine regulated on activation, normal T cell express and secrete marker and lower levels of PAI-1, soluble intracellular adhesion molecule, and soluble vascular cell adhesion molecule than pediatric patients who died. Adult patients had similar levels of these analytes regardless of outcome [59,60].

#### Neurocognitive pathology

EBOV infection includes symptoms such as headache, encephalitis, meningitis, cerebral edema, and seizures, which indicate the virus involvement in pathology of the central nervous system (CNS). Owing to the high mortality rates associated with Ebola virus infection, studies of the long-term neurological and neurocognitive effects are virtually non-existent. Nevertheless, news reports have suggested that EBOV-survivors continue to experience complications such as headache, weakness, fatigue, sensory changes, and inflammation of certain organs. While there is evidence to suspect CNS complications among survivors, this may go unrecognized or unreported [61]. Considering our current knowledge of Ebola viral mechanisms, reported complications from long-term survivors, and knowledge of the adverse effects of pro-inflammatory cytokines on neuronal function, three potential contributors to ongoing CNS dysfunction in EBOV survivors are considered below:

1. persistent inflammation of the CNS through the release of cytokines and chemokines and recruitment of infected monocytes,
2. disruption of the blood brain barrier (BBB), which increases its permeability and deregulation of tight junction proteins, and
3. acquired neurological insults resulting from Ebola virus infection.

#### Inflammation and CNS

Ebola virus infection causes the initial activation of monocytes/macrophages, which in turn releases cytokines that target the vascular system, particularly endothelial cells [62]. While transient innate immune responses in the form of cytokines are beneficial to the host, the same essential spectrum of cytokines leads to deregulation of homeostatic mechanisms, destruction of host tissues and apoptosis [63]. Transmigration of the Ebola virus into monocytes/macrophages triggers the increased synthesis of TNF-α, which induces Ebola virus hemorrhagic fever and contributes to lymphoid cell apoptosis and marked inhibition of interferonα/β. Infection also leads to the release a variety of pro-inflammatory proteins that include IL1β, IL6, IL8, IL15, IL16, the chemokines macrophage inflammatory protein (MIP-1)-α and -β, monocyte chemotactic protein 1 (MCP1), macrophage colony-stimulating factor (M-CSF), macrophage migration inhibitory factor (MIF), IFN-γ-induced protein 10 (IP-10), and eotaxin to name a few [64]. However, monocyte-derived dendritic cells do not secrete proinflammatory cytokines of TNF-α, IL1β, IL6, IL10, or IL2, IFN-γ, or IL12 after infection with Ebola virus, though there are higher levels of certain chemokines (notably IL8 and MCP1) [65]. Taken together, these data indicate that monocytes/macrophage subpopulations release large quantities of both cytokines and chemokines, whereas there is a selective negative effect of Ebola virus on the induction of the pro-inflammatory cytokines by dentritic cells.

In the brain, receptors for IL1β, IL6, TNF-α, IFN-γ and MCP1 have been the most widely studied in relation to neuropsychiatric and neurological disorders. Cytokines have both atherogenic and prothrombotic effects, which may directly influence neurological events such as ischemic stroke or vascular dementia. EBOV produces coagulation defects via cytokine production that directly influence the coagulation cascade. IL1β, IL2, IL6, and TNF-α have been directly implicated in the stimulation of the coagulation system. Abnormalities of the brain coagulation system have been implicated in traumatic brain injury [66].

In rodent brains, the hippocampus and hypothalamus (areas responsible for learning/memory and body homeostasis, respectively) have the highest concentration for receptors of IL1β, IL2, IL6, and TNF-α, which can become up-regulated during peripheral immune activation [67]. IL1β is also highly concentrated in cerebrovascular endothelium, and increase after excito-toxic injury, LPS challenge, mechanical damage, and ischemia [68]. Injection of IL-1β into specific brain regions has been found to exacerbate neuronal damage, regardless of the primary insult (e.g., trauma, ischemia). In response to injury, IL1β induces microglial proliferation and microglial expression of IL6, though experimental depletion of IL-6 has been found to protect against the adverse effects of IL1-β and TNF-α on working memory [69]. These findings suggest that IL-6 may exacerbate the effects of IL1β and TNF-α.

IL6 plays a critical role in adult neurogenesis [70], which is altered in many neuropathological situations like stroke, *status epilepticus*, mechanical damage, and Alzheimer, Parkinson and Huntington diseases, which all share inflammation in common [71,72]. IL6 has been thought to exert neurotrophic effects by preventing oxidative stress and apoptosis. One study found that inhibiting IL6 or its downstream JAK/STAT signaling pathway in the orbitofrontal cortex impaired reversal learning, suggesting that basal IL6 and JAK/STAT signaling facilitate cognitive flexibility [73].

TNF-α is involved in regulating synaptic transmission and plasticity in the CNS. Elevations in TNF-α, usually produced by astrocytes and microglia, are associated with several neurological disorders, and results in an inhibitory effect on glutamate transporters, resulting in increased glutamate concentration in the CNS [74]. This affects cognitive processes such as learning and memory [75,76], sleep [77], food and water intake [78].

MCP-1 is a β-chemokine that is expressed during inflammation and which, upon activation of its receptor, CCR2, can induce chemotaxis of monocytes to inflammatory sites generated by injury and infectious events [79]. MCP-1 has been identified as the most potent activator of macrophages in comparison to other monocyte chemo-attractants, including RANTES, MIP-1α, MIP-1β, MCP-2β and MCP-3 [80,81]. MCP-1 levels in the brain and cerebrospinal fluid (CSF) are elevated in neurological conditions such as AIDS dementia, cerebral inflammation [82-88].

Hence inflammation, mediated in part by chemokine activity and the release of proinflammatory cytokines, contributes to the breakdown of the brain microvascular endothelial cells that constitute the BBB (Barkhordarian et al., in press) increasing the potential for continued viral invasion into the CNS. While most studies have focused on the inflammatory response of microglia and astrocytes [89-91], perivascular cells also play a key role in brain inflammation [92]. CNS pericytes are involved in recruitment of peripheral cells to the brain, which may directly produce neuronal damage or promote microglial inflammation [93,94].

#### Blood Brain Barrier (BBB) disruption

The BBB separates the brain from the circulatory system and protects the central nervous system from potentially harmful chemicals while regulating transport of essential molecules and maintaining a stable environment [95,96]. Specialized endothelial cells that line brain capillaries and transduce signals from the vascular system and brain form its structure. Both the structure and function of the BBB is dependent upon the complex interplay between the different cell types (such as the endothelial cells, astrocytes, and pericytes), and the extracellular matrix of the brain and blood flow in the capillaries [97]. Three sites have been identified with a physical barrier via tight junctions that include the brain endothelium (forming the BBB), the arachnoid epithelium (forming the middle layer of the meninges), and the choroid plexus epithelium, which secretes CSF [91].

The BBB contains specialized endothelial cells that are attached through tight junctions and adherence junctions, which function to separate the CNS from the circulation and restrict and prevent blood-borne molecules and peripheral cells from entering the CNS [98]. Further, tight junction proteins also provides the BBB with two functionally distinct sides: the luminal side facing the circulation and the abluminal side facing the CNS parenchyma, which are highly sensitive to major cytokines produced during such a response, including TNF-α, IL1-β, and IL6 [99]. Inflammatory cytokines of the TH17 and TH9 families (*vide infra*) may contribute in opening selected “gates” through the BBB (Barkhordarian et al., Bioinformation, in press).

Experimental studies of human cultured embryonic cells have demonstrated that virus-like particles (VLPs) consisting of the Ebola virus matrix protein VP40 and GP1,2 activated endothelial cells and disrupted barrier function. This was further enhanced by TNF-α, which is known to induce a long-lasting decrease in endothelial cell barrier function and is hypothesized to play a key role in Ebola virus pathogenesis [100].

Other viruses, including the Human Immunodeficiency Virus (HIV), produce viral proteins such as gp120, Tat, and Nef released from infected cells, which lead to increased expression of adhesion molecules such as intercellular adhesion molecule (ICAM)-1 and endothelial adhesion molecule [101]. These molecules facilitate the transport of HIV within these infected macrophages by promoting their ability to bind and migrate across the BBB, although cell-free virions are also thought to be able to cross the BBB due to changes in tight-junction formation [102,103].

#### Neurological syndromes

In 1967, cases of hemorrhagic fever occurred in Marburg. The Marburg virus (MBGV) and EBOV, as noted above, are both members of the family *Filoviridae*, and are very similar in terms of morphology, genome organization, and protein composition [104]. The pathology of Marburg disease was investigated from gathering tissues from brain, spleen and liver from those infected and have been used to make inferences about the pathology of Ebola virus. Brain tissue derived from those infected with the Marburg virus were noted for brain swelling, increases in vascular permeability, associated reduced effective circulating blood volume, and interstitial edema in the brain. The pathologic alterations found in Ebola virus infection appear to share similar features to those found in Marburg virus infection, though an absence of comprehensive comparative studies remain.

EBOV has been found to infect the meninges. Most viral infections that involve the meninges can manifest into progressive neurologic disorders. Clinical findings reflect disease progression and the specific areas of CNS involvement, which is determined by viral tropism [105]. For example, polioviruses preferentially infect motor neurons, mumps infects epithelial cells of the choroid plexus [106]; however, the tropism of filoviruses remain unclear [107].

Encephalitis due to infection, post-infections encephalomyelitis (which may occur after measles or Nipah virus encephalitis) and conditions such as post-poliomyelitis syndrome, which some believe is a persistent manifestation of poliovirus infection, are examples of neurological syndromes that can occur with Ebola virus invasion. Symptoms that signal encephalitis due to infection of the meninges include sudden fever, headache, vomiting, heightened sensitivity to light, stiff neck and back, confusion and impaired judgment, drowsiness, weak muscles, a clumsy and unsteady gait, and irritability. Even if the blood brain barrier is not impaired, neuroinflammatory mediators crossing the BBB may result in neurotoxic effects due to the accumulation of free radicals from the brain’s cytotoxic response [108]. Symptoms that require emergency treatment include loss of consciousness, seizures, muscle weakness, or sudden severe dementia. Chronic meningitis from *Cryptococcus* usually develops among patients with compromised immune systems (e.g., elderly, AIDS patients). CT or MRI is usually recommended first, before the lumbar puncture [109]. Encephalitis and meningitis can cause cerebral edema, and cerebral edema among those infected with Ebola virus has been reported. Cerebral edema is a dangerous condition where the brain’s water content rises, causing the pressure to rise in the skull. Swelling can disrupt oxygen supply as the blood vessels become squeezed. Cerebral edema is a medical emergency that can even lead to death as brain cells become damaged and die. Global cerebral edema is a powerful and consistent predictor of cognitive dysfunction [110]. However, determining the long-term cognitive consequences of these cases are rare, as they are often associated with high mortality rates.

In brief, while the research on the direct effects of EBOV on CNS is limited, we can only draw from knowledge about viral entry, viral propagation, and multiple organ failure about the potentially profound effects on the central nervous system. Survivors are at increased risk for cognitive impairments. As with most neurological conditions, prognosis for cognitive outcomes will be based upon the timing between infection, diagnosis, and treatment.

#### Immunopathology

Infection with EBOV leads to aggressive and highly lethal hemorrhagic fever in humans. Its virulence is associated with a variety of processes and events that engage, and subsequently blunt cellular immune surveillance. Initial immune responses include a rapid rise in pro-inflammatory cytokines (e.g., IL6, TNF-α), which trigger a relatively short-lived initial burst of fever and inflammation often disregarded by the patient, as well as cellular immune migration factors (e.g., IL8). Soon into the immune surveillance response, which commences immediately after infection, a slower process on cellular pathology ensues, which includes myeloid cell and endothelial cell infection and cytopathology. Consequential to this second phase are both a sharp rise in fever, and loss in vascular integrity, which leads to increased permeability of blood vessels with transudates increasingly rich in micronutrients, red blood cells and eventually white blood cells. Deficiencies in specific and nonspecific immune-driven antiviral responses result in unrestricted EBOV replication and dissemination in the host, which together lead unavoidably - unless prompt intervention is engaged to prevent EBOV binding to target cells or block its replication within infected macrophages and dendritic cells, and/or to counter the physiological collapse due to dehydration secondary to heavy bleeding and violent diarrhea - to death within 14 days following the appearance of EVD symptoms.

In brief, EBOV actively subverts both innate and adaptive immune responses, and triggers harmful inflammatory responses that inflict direct tissue damage. The organism is ultimately overwhelmed by a combination of inflammatory factors and virus-induced cell damage, particularly in the vasculature, often leading to death from liver and kidney failure, complicated by septic shock [111].

The *filoviridae* family of viruses, including EBOV, causes severe impairment of innate and adaptive antiviral cellular immune responses, in part as a result of virally encoded immune antagonists, which render the host incapable of mounting effective innate or adaptive immune responses. Type I interferon, which include IFN-α, IFN-β, IFN-κ, IFN-δ, IFN-ε, IFN-τ, IFN-ω, IFN-γ and IFN-ζ, are impaired by EBOV. Of these IFN-α and IFN-β are secreted by lymphoid and myeloid cells, and play an important role the anti-viral cellular immune response. Certain filoviral-encoded components target IFN-α and IFN-β responses, and blunt the immune-mediated suppression of viral replication, thus favoring and Ebola pathogenesis. Case in point, EBOV VP35 facilitates immune evasion of EBOV by inhibiting the phosphorylation of interferon regulatory factor 3 & 7 (IRF3/7) by TANK-binding kinase 1 (TBK1) and IkappaB kinase epsilon (IKKε) (TBK1/IKKε) kinase activity jointly as it acts to sequester the EBOV viral RNA from detection by retinoic acid inducible gene-I (RIGI)-like receptors. As such, EBOV (and MRGV) VP35 acts as an interferon inhibitory domain (IID). Moreover, EBOV VP24 inhibits nuclear translocation of activated STAT1 by karyopherin-α, thus rendering cells refractory to IFNs [112,113].

EBOV-like particles (eVLP), composed of the EBOV glycoprotein and matrix viral protein (VP)-40 with a lipid membrane, are a highly efficacious method of immunization against EBOV infection. The exact requirements for immunity against EBOV infection are poorly defined at this time. Vaccination of BALB/c or C57BL/6 mice with eVLPs in conjunction with QS-21 adjuvant resulted in mixed IgG subclass responses, a Th1-like memory cytokine response, and protection from lethal EBOV challenge. Further, this vaccination schedule led to the generation of both CD4+ and CD8+ IFN-γ producing T cells recognizing specific peptides within glycoprotein and VP40. The transfer of both serum and splenocytes, but not serum or splenocytes alone, from eVLP-vaccinated mice conferred protection against lethal EBOV infection in these studies. B cells were required for eVLP-mediated immunity to EBOV because B cell-deficient mice vaccinated with eVLPs were not protected from lethal EBOV challenge. CD8+, but not CD4+, T cells are absolutely required for eVLP-mediated protection against EBOV infection. Further, eVLP-induced protective mechanisms appear to be perforin-independent, but IFN-γ-dependent. Taken together, both EBOV-specific humoral and cytotoxic CD8 + T cell responses are critical to mediate protection against filoviruses following eVLP vaccination [114].

In brief, the first telling symptoms of incipient EVD include high fever and bleeding from the orifices. As dendritic cells and macrophages succumb to EBOV infection, the cellular immune surveillance mechanism is progressively impaired, and virus titter rises, as does EBOV-caused cyto-and physiopathology. Immune evasion events of EBOV include:

- inhibition of innate immunity that precipitates down-regulation of type I interferons, as well as IFN-γ,
- EBOV epitope masking,
- subversion of the adaptive humoral immunity, and
- secretion of truncated forms of the viral glycoprotein (e.g., GP, ssGP) [115].

It is possible and even probable that EBOV-infected dendritic cells and macrophages soon invade specific organs, and establish themselves as EBOV-infected resident myeloid cells. Case in point, renal myeloid cells represent a constitutive, extensive and contiguous network of local immunity cells that provide sentinel immune surveillance. They contribute to induce and regulate inflammatory responses to protect the kidney from infection. They are a perfect example of local immunity, as similar sentinel myeloid cells are found in every organ to play key factors in the initiation and propagation of renal disease, as well as essential contributors to subsequent tissue regeneration, regardless of etiological and pathogenesis mechanisms [116].

Indeed, dendritic cells and macrophages, in part because of their high degree of motility, play a critical role in the cross-talk between systemic and local immunity, including between the central nervous system and mucosal immunity – that aspect of local immunity is most likely to be severely affected during EBOV infection. HIV is another RNA virus that infects myeloid cells: HIV+ dimCD4 + macrophages leave the systemic immunity environment, to infiltrate in the local immunity of the intestinal mucosa, in so doing, contribute to promote local inflammation and injury to the intestinal lining, contributing to loss of the resident intestinal flora and heavy diarrhea [117]. More generally speaking, HIV can escape from systemic immunity assessments simply by becoming hidden in macrophages and dendritic cells that transmigrate from the systemic compartment to organs and lymph nodes, such that lymphadenopathy is a clinically relevant sign and important guiding tools for detecting hidden HIV in asymptomatic individuals [118,119].

Evidently, we do not know if this is the case in EBOV infection as well because the pathology of EVD is so fulminant and lethal. However, as the number of EBOV+ survivors will continue to increase, it will behoove us to be more prudent and less euphoric in our statement that such and such a patient is “cured” from Ebola: rather, we ought to consider the likelihood that EBOV, like HIV, could become hidden in the micro-environment of local immunity, thus becoming undetectable as virions or viral particles in the systemic circulation. This eventuality should be particularly apparent when “cured” patients retain high titers of circulating anti-EBOV antibodies, or certain symptomatologies (e.g., severe headaches, confusion, fever).

EBOV causes highly lethal hemorrhagic fever that leads to death in up to 90% of infected humans, depending on the Ebola virus species. Like many other infections, EBOV induces massive lymphocyte apoptosis, which is thought to prevent the development of a functional adaptive immune response. In a murine experimental model of EBOV infection, data described an increase in expression of the T cell activation-migration-maturation marker CD44 in CD4+ and CD8+ T cells. CD44 is a cell-surface glycoprotein involved in cell–cell interactions, cell adhesion and migration. The percent and absolute numbers of the CD4 + CD44+ and CD8 + CD44+ subpopulation sharply increase in the later phase of EBOV infection, preceding death. In many instances, this event is accompanied by a dramatic rebound of lymphocyte numbers in the blood, which however appear to be apoptotic lymphocytes, suggesting that local (i.e., spleen) and systemic cellular immunity might be characterized by considerable unproductive lymphocyte activation, and consequential programmed cell death in the later stages of EBOV infection. Data have indeed shown that, despite significant lymphocyte apoptosis, a functional and specific, albeit insufficient, adaptive immune response is made in lethal EBOV infection and could be protective upon transfer to naive infected recipients. These findings may spark new immune-based therapeutic strategies to control EBOV and EVD [120].

EBOV infection induces massive lymphoid and myeloid apoptosis. Data have established that EBOV-induced lymphocyte apoptosis *in vivo* occurs via both the death receptor (extrinsic) and mitochondrial (intrinsic) pathways. Inhibiting lymphocyte apoptosis during EBOV infection fails to improve animal survival rates [121], indicating that the immunopathogenesis induced by EBOV occurs upstream from T cell activation. Clearly, one must surmise from these findings, taken together, that EBOV infection of antigen-presenting cells, such as macrophages and dendritic cells, must alter the plasma membrane structure of the host cell, such that the T cell, while recognizing the non-self/self complex, fails to find a functional CD80 (B7.1) and CD86 (B7.2) receptor on the membrane of the EBOV-infected antigen presenting cell for its CD28 co-activating membrane moiety. Alternatively, the T cell inducible costimulator (ICOS) might fail to find an integral form of its ligand, B7RP1, of the EBOV-infected macrophages and dendritic cells. The hypothetical model we propose here is grounded on our current understanding that costimulatory signals, such as CD28 and ICOS are critical for the establishment and maintenance of systemic and local cellular and humoral immune surveillance, and rests on previous observations in a related model that demonstrated that the impairment in the B7 family-ligand costimulatory pathways may promote tumor immune evasion by providing a negative regulatory signal equivalent to T cell apoptotic cell death [122]. Indeed, this paradigm has led to the development of a variety of immunotherapies targeting costimulation pathways [123-125]. It follows that future clinical trials in EBOV+ patients may require testing immunotherapeutic interventions such as these as preventive or palliative means of blunting EVD.

### Clinical interventions

Currently the diagnosis of Ebola is difficult [61,126,127], and there are no specific vaccines or medicines (such as antiviral drug) that have been proven to be effective against Ebola. Symptoms of Ebola are treated as they appear. The following basic interventions, when used early, can significantly improve the chances of survival:

- Providing intravenous fluids and balancing electrolytes
- Maintaining oxygen status and blood pressure
- Treating other infections if they occur

Timely treatment of EVD is important but challenging since the disease is difficult to diagnose clinically in the early stages of infection. Because early symptoms such as headache and fever are not specific to ebolaviruses, cases of Ebola may be initially misdiagnosed, as noted above [128].

#### Vaccines

We discussed elsewhere the main difficulties in developing vaccines for HIV [129]. Similar concerns arise for related RNA viruses, including the filoviruses, and EBOV in particular. As for anti-HIV vaccines [129], the most promising anti-EBOV vaccine candidates are DNA vaccines raised against the EBOV cDNA, vaccines derived from adenovirus-EBOV conjugates, or vaccines directed against certain EBOV components, such as VLPs. Using mRNA extracted from bone marrow of Ebola survivors, human monoclonal antibodies against Ebola virus surface protein have been experimentally produced and now raise the hope for the development of a safe vaccine [130]. However, as of November 2014, no vaccine was approved for clinical use in humans, or even developed, tested for toxicity, and distributed for that initial purpose.

The complexity in developing a EBOV-specific vaccine is in part the reason why no licensed vaccine is available yet. It is also the case that prior outbreaks, considering their size and relative containment, failed to evoke the response of the national and international scientific community in pursuit of an anti-EBOV vaccine [131,132]. Even in the course of the present epidemic, the call for the development of an effective vaccine protective against Ebola was relatively subdued until EBOV+ patients were identified on European and US soil. Now, Ebola vaccines are undergoing active investigation, particularly in light of a more complete understanding of the genomic and proteomic properties of EBOV [133].

By all accounts, a vaccine is necessary to halt the present Ebola epidemic. The two most advanced candidates have recently entered safety and efficacy trials. Should they prove non-toxic and efficient in triggering the needed immune response, then WHO might recommend bypass of the usual careful screening and testing process, and immediate utilization in what would amount to phase III efficacy tests in Liberia, Guinea, and Sierra Leone, and possibly in Western Europe and the US as well. Case in point, Canada’s National Microbiology Laboratory developed an experimental vaccine that combines fragments of Ebola virus with the non-fatal adenovirus. The vaccine appears promising, and testing in the US (Walter Reed Army Institute of Research) has begun, and will follow presently with testing in Western Europe and Africa. However, the safety and effectiveness of the Canadian vaccine in humans remains unknown at this time. Similarly, simultaneous phase I trials of an experimental vaccine (NIAID/GSK), based on a modified chimpanzee adenovirus conjugated to portions of Zaire and Sudan EBOV, and developed jointly by GlaxoSmithKline and NIH, commenced in September 2014. If this phase is completed successfully, then a stockpile of 10,000 doses of the vaccine could be fast tracked for use in West Africa and world-wide, perhaps as early as early 2015, although full validation studies will not be completed by then.

GlaxoSmithKline website reported in mid-October that “…“*development of the vaccine candidate is progressing at an unprecedented rate, with first phase 1 safety trials with the vaccine candidate underway in the USA, UK and Mali, and further trials due to start in the coming weeks*…”. As an inevitable *sequela* of this fast-track process however, difficult bioethics questions are emerging in the rationale that would determine who would be tested with these vaccines, vs. others who would be left to face a quasi certain lethal fate [134].

#### Pharmacological interventions

Without an approved vaccine or treatments, Ebola outbreak management has been limited to palliative care and barrier methods to prevent transmission. These approaches, however, are increasingly inefficient as the epidemic grows exponentially. Certain drugs have been approved for use in Ebola patients, and characteristically inhibit filovirus cell entry, such as amiodarone, dronedarone and verapamil [135].

Alternative pharmaceutical interventions, such as the combination of monoclonal antibodies optimized from two previous antibody cocktails (ZMapp, produced by Novartis, Mapp Biopharmaceutical, and others), appears to rescue 100% of rhesus macaques when treatment is initiated up to 5 days post-challenge. High fever, viremia and abnormalities in blood count and blood chemistry are relieved following ZMapp intervention. Advanced disease, as indicated by elevated liver enzymes, and ZMapp reverses mucosal hemorrhages. In brief, ZMapp exceeds the efficacy of any other therapeutics described so far, such as RNAi-based TKM-Ebola, or antisense based AVI-7537) [136-138].

ZMapp outcomes were so promising to warrant further development of this cocktail for clinical use. Indeed, it was administered to the two US medical workers Dr. Kent Brantly and Nancy Writebol, who had contracted EVD in Liberia, were transported to US hospital, and, following ZMapp treatment, recovered from Ebola. ZMapp is certainly not the “miracle drug”, however, as the case of the Spanish priest who got infected with EBOV in Liberia, and eventually died of Ebola in Madrid a few days into his treatment regimen with ZMapp demonstrates. Teresa Romero, the nurse who treated him, contracted the disease and was the first case of Ebola contracted and diagnosed outside of the African continent, as discussed above.

### Toward novel interventions

#### Cellular immunity

Case reports of acutely ill patients with EVD showing improvement with the transfusion of plasma from EVD survivors may seem at odds with the idiotypic antibody network model of immunoglobulin regulation, first advanced four decades ago [139]. However, in the case of EBOV, which is know to disrupt cellular immunity upstream of T cell activation and differentiation into lymphocytes capable of either the TH1 or TH2 pattern of cytokines, it appears plausible that replenishment of immunoglobulin may actually be protective, rather than nefarious to the humoral immune response, at least during a certain window in time of immunopathology.

The microenvironment of organisms is greatly dependent on the intricate, fluid relationships that exist between the different subpopulations of T CD3+ cells, and the pattern of cytokines they produce. Murine immunology has revealed two principal clones of T cells characterized by their secreted cytokines, Thelper1 (Th1) and Th2. Human immunology studies uncovered similar patterns of cytokines, which however could not be cloned as in the murine system for obvious reasons. Therefore, and to recall the murine model, these patterns are referred to as TH1 and TH2 T cell subpopulations. Whereas the human TH1 cytokines (e.g., IL2, INF-γ, IL12) predominantly favor T cell activation, proliferation and maturation for cellular immunity toward parasites, virally infected cells and tumor cells, the TH2 cytokine profile (e.g., IL4, IL5, IL10) favors the activation, proliferation and maturation of B cells and enhance humoral immunity and the production of antibodies. A third population was soon characterized both in the murine and in the human systems, which down regulated T cells responses, and tended to blunt cellular immunity: this regulatory T cell subpopulation (Tregs) was characterized by tri-immunofluorescence flow cytometry to express either CD4 or CD8, to be mostly constituted of activated cells expressing the α chain of the IL2 receptor, CD25, and the characteristic key signature of Tregs, FoxP3. Further characterization revealed that, depending upon the microenvironment, TH1 populations might engender a TH17 subpopulation, whose cytokine profile (e.g., IL17-A, IL-17 F, TNF-α, IL22, IL23, and IL9 in low concentrations) lends to a state of sustained T cell-driven inflammation seen in in autoimmune diseases and allergic reaction, or TH2 cells may generate TH9 subpopulations characterized by elevated levels of IL9 and IL10. Recent data indicate that Tregs play a critical role in directing and regulating the dynamic plasticity of the balance of TH1/TH2, and of the intimately related TH17/TH9 subpopulations [140-144].

TH1/TH2 and TH17/TH9 relationship is pivotal in maintaining the efficiency of cellular immune surveillance. Should the hypothesis that EBOV impairs the host’s cellular immunity by altering the Tregs-mediated regulation of TH1, TH2, TH17 and TH9 plasticity be proven true, then novel immunotherapies could be designed and tested on EVOV+ patients directed specifically at restoring the physiological homeostasis in TH1, TH2, TH17 and TH9 cytokines.

Case in point, IL23 is important for the expansion of TH17 cells and thus the production of IL17-A and IL9. However, IL23 is not necessary for the initial differentiation of TH17 cells but it maintains and stabilizes the TH17 lineage [143], which itself is the principal driver for the maintenance of TH17-mediated T cell-induced and -sustained inflammation. By promoting Tregs, the expression of IL23-receptor is abrogated, which leads to the inhibition of IL23 production, and consequentially TH17-driven events are blunted, As a result, a plausible increase in TH9 cells should occur due to the strive for balance of TH17 and TH9 cells in the immune system. It is also the case that IL6 and low concentration of induces IL-23 receptor expression and high concentration of TGF-β inhibits IL23 receptor and mediates a dynamic conversion between TH17 and Tregs by inducing Foxp3+ transcription factor [142-144].

In brief, cellular immune surveillance is a dynamic and fluid system that is driven by a finely balanced and delicately regulated equilibrium of cytokines. The microenvironment of the organism, which is profoundly altered in the case of EBOV infection and the consequential physiological breakdown in EVD, dictates and regulates whether or not there is a predominant TH1 and TH17, or TH2 and TH9 pattern of cytokines. It follows that novel immunotherapeutic modes of combating EBOV immunopathology may simply lie in rectifying the cytokine relationship and the fluidity of the T cell subpopulations that are driving these cytokine patterns.

Reiterating the concepts made above, the observation that seven of eight patients with Ebola survived after receiving a transfusion of plasma donated by individuals who had previously survived the infection is coherent with both our current understanding of cellular immunity, and of the immunopathology mediated by EBOV. In brief, intravenous antibodies appear to be protective, and therefore, WHO has approved the use of convalescent serum and plasma to treat patients with Ebola.

EBOV-infected macrophages and dendritic cells produce pro-inflammatory cytokines, chemokines and tissue factor, attracting additional target cells and inducing vasodilatation, increased vascular permeability and disseminated intravascular coagulation. However, they appear incapable of restricting viral replication, in part because of impaired interferon responses (*vide supra*), but also because of other cytopathic mechanisms yet unclear. Consequently, EBOV spreads throughout the body via these cell populations, and causes multifocal sites of necrosis and septic shock-like syndrome. Massive apoptosis of natural killer and T cells accompanies these events, as noted above, and this further impairs immunity [127,145,146]. These findings suggest that the virus causes profound alterations in macrophage biology, and that perhaps modifying host responses via correcting EBOV-caused macrophage defects could become an effective alternative therapeutic strategy in the treatment of Ebola.

Macrophages are myeloid derivatives that mature from monocytes and that process foreign materials by phagocytosis, a process that has evolved in vertebrate immunology to recognize pathogens and damaged tissues through Toll receptors, as well as pathogen-associated molecular patterns (PAMP) and damage-associated molecular patterns (DAMP) [146-155]. In general terms, macrophages either turn on their killing program and “fight” against an invading pathogen and defend against subtle variations of self and to develop and engage specific T cell-mediated immune responses, or they engage in a “fix” repair and remodeling program. Macrophages either elicit responses that include nitric oxide and oxygen radical production, which have been described as a M1-type macrophage response, or production of factors that promote proliferation, angiogenesis, and matrix deposition, which together represent a M2-macrophage response [147,149,152,156,157].

Macrophage-like cells have varied tissue distributions, and have different names depending on their anatomical sites. In the central nervous system, macrophages are called microglial cells whereas hepatic macrophages are referred to as Kuppfer cells, in the lungs they are recognized as alveolar macrophages whereas skin macrophages are the Langerhans cells [147,152]. Depending on their histologic and microenvironment, macrophages have distinct functions, so that Langerhans cells, for example, are most often engaged in encountering damage signals due to wounds and act predominantly in an M1-type response, whereas alveolar macrophages, for instance, predominantly engulf inhaled materials and work predominantly in an M2-fix modality.

M1 and M2 are oversimplified descriptions of the complex processes brought about by macrophages in their function as director of the innate immunity orchestra; but, the model provides a useful dichotomous functional classification that segregates the macrophage toxicity from its repairing activity at the cellular and histological level. In brief, M1 and M2 refer to two different, actually opposing and balancing states and activities of macrophages: in the M1 stage, cells predominantly produce cytotoxic levels of nitric oxide, whereas cells at the M2 stage largely secrete cell- and tissue levels of ornithine [157,158].

Macrophages actively metabolize arginine either to nitric oxide and citrulline via the inducible nitric oxide synthase pathway (iNOS) (M1 physio-toxic profile), or to ornithine and urea via the arginase pathway (M2 physio-repairing profile). Nitric oxide is the primary effector molecule used by macrophages to kill pathogens or eliminate host cells [159-163]. By contrast, ornithine is a precursor of polyamines, which are required for DNA replication [164,165], as well as of L-proline, a major component of collagen that is required for tissue repair and serves as a matrix promoting cell replication [166,167]. The M1 macrophage pattern influences TH1 cytokines, including IL-12, which drive T-cell sustained inflammation (i.e., TH17), activation, proliferation and maturation; by contrast, macrophages in the M2 state tends to favor TH2 patterns of cytokines, which support humoral immunity, including B cell proliferation, maturation and secretion of antibodies [148,152,153].

One among the most powerful stimuli for iNOS, and therefore for inducing M1 macrophages is IFN-γ. Nitric oxide and citrulline can be generated during both innate and adaptive immune responses since T lymphocytes and natural killer cells, upon activation, produce large amounts of IFN-γ [147,148,154,155,168-171]. IL12, a TH1 cytokine produced by dendritic cells and macrophages, stimulates IFN-γ and thus contributes to driving forward M1 macrophages [163,165,171,172]. By contrast, TGF-β1 stimulates the arginase pathway, and is potent inhibitor of iNOS, such that the constitutive production of TGF-β1 and nitric oxide by macrophages is inversely correlated: TGF-β1 serves as an autocrine regulator for iNOS, an endogenous switch, as it were, that significantly contributes to determining a M1 or an M2 stage [157,163,169,173-182]. IL4 induces TH2 responses, while suppressing TH1 patterns of cytokines, and together with IL13 contributes to inhibiting the production of nitric oxide, inducing arginase and favoring the transition to the M2 state of macrophage function [154,183,184].

In a classic pattern of cellular immune surveillance, pathogens and cyto-histological damage, including cancer and virally-infected cells, are detected through PAMP and DAMP within hours; shortly thereafter, a set of signals are produced by resident macrophages, which are dedicated to act as M1 macrophages within a day of the insult. Simultaneously, levels of IFN-γ produced by immune immunity cells rise, which further elevate the M1 response [149-151,156,171,185-190].

As the pathogen invades lymph nodes, the lymph nodes antigen-presenting cells (i.e., dendritic cells) provoke, by their presenting the antigen, the clonal proliferation of antigen-specific T cells [191]. During a M1 macrophage response, IL12 and IL23 are secreted, which signal T cells to elicit a TH1 response, including high levels of IFN-γ. The ongoing M1 response is therefore sustained, and TH17 activation ensues further amplifying the M1 cytotoxic and histopathologic response [191]. By contrast, when the pathogen is contained by the M1 pattern of response, then M2 state macrophages engender a pattern of cytokine that is associated TH2 pattern of cytokines. This includes IL10 that shuts down the TH1 cytokine response, as well as IL4 and IL13 that promote antibody production for the removal of the pathogen, and TGF-β-1 that further induces arginase for cell and tissue rebuilding and repair. Therefore, to a INF-γ/TGB-β-1 balance and a TH1/TH2 balance seem to correspond a M1/M2 balance and a tissue destruction by virtue of excessive nitric oxide and related cytotoxic compounds vs. a tissue regeneration modality resulting from arginase-mediated production of polyamines for DNA repair and L-proline and ornithine for cell and tissue repair [191-193].

If the hypothesis can be brought forward that EBOV selectively alters the M1/M2 balance because it is tropic to macrophages and dendritic cells – a hypothesis that could be tested relatively simply by *in vitro* manipulations followed by tri- or tetra-immunofluorescence flow cytometry – then, the inference can be brought forward that EBOV may contribute to drive and sustain an M1 state simply because of the chronic depletion of ebolavirus-infected macrophages and the need to generate new myeloid derivatives to combat the pathogen. Consequently, a state of super-induced iNOS could ensue – a hypothesis equally testable with biopsy specimens from Ebola patients – and the associated profound cellular and physiological toxicity, which could contribute to the system collapse that manifests in the advanced stages of EVD. Novel immune-based interventions for Ebola patients might therefore involve modulating the M1/M2 balance by attenuating M1 responses and favoring an M2 macrophage state.

Indeed, macrophage-directed interventions include 2 pharmacotherapeutic drugs directed at impairing macrophage survival [194,195], although it is not clear if it may specifically target M1 or M2. Research shows, at least in the context of central nervous system microglia and invading macrophages, that NF- κB p50 is a key redox-signaling mechanism regulating the M1/M2 balance, which preferentially drives M1 responses [196,197].

### Microparticles and micronutrients

The deregulation of coagulation cascade due to EBOV hemorrhagic fever is a syndrome that causes disseminated intravascular coagulation, which leads to micro-thrombi and related fibrin deposition in the microvasculature. Multiple organ failure ensues, as was outlined above. At the molecular level, the overexpression of the extrinsic tissue factor (TF) trans-membrane glycoprotein is responsible for fibrin deposition. That TF is expressed, not by endothelial cells, but by myeloid cells is indicative of putative TF alterations in EVD since, as noted, EBOV infects monocytes and macrophages. Data obtained in experimentally EBOV-infected macaques show altered TF expression on microparticles, small vesicles (0.2–1 μm) derived from cell plasma membranes and acting as intermediate messengers, probably of myeloid cell origin. Microparticle TF, detected by days 4 and 5 post-infection, were shown to have an important pro-coagulant role. Taken together, these preliminary studies suggest that chemotherapeutic strategies aimed at controlling overexpression of microparticle TF may prove to be an effective intervention to counter EBOV-induced EVD hemorrhagic fever [198].

Selenium is an essential micronutrient in human homeostatic physiology. Selenium acts as an essential component of the unusual amino acids, seleno-cysteine and seleno-methionine. In states of selenium deficiency, cells cannot synthesize seleno-proteins, which act not only in selenium transport, but also as essential auto-oxidants to maintain cell health and to prevent cytopathic manifestations. Seleno-proteins also maintain physiologic balance through their anti-inflammatory properties. Selenium is consumed as part of a normal diet, or as a dietary supplement [199]. Seleno-cysteine proteins contain selenium in their active site in the form of their 21st amino acid seleno-cysteine, which is encoded by an in-frame UGA stop codon [200]. Several UGA codons lie in Ebola genome and some seleno-cysteine insertion sequences (SECIS), in particular stem-loop structures, were predicted in mRNAs analyses. It is possible and even probable that EBOV UGA codons may encode seleno-cysteine, thereby depleting the selenium available to the host cell, impairing eukaryotic seleno-protein translation, and prompting lipid peroxidation and consequential cellular damage. Secondarily, selenium depletion may lead to both enhanced thrombosis and decreased immune surveillance processes, acting in concert to worsen hemorrhagic symptoms [201]. It follows that novel nutritional paradigms aimed at replenishing selenium might be desirable for maximum protection against, and recovery from EVD.

### MicroRNA

MicroRNA is a small non-coding RNA molecule, which is usually smaller than 20–22, and which functions to silence RNA post-transcriptional regulation of gene expression generally by forming base-pairing with complementary sequences within mRNA molecules. Animal miRNAs are usually complementary to a site in the 3’-UTR, and function in gene regulation principally by inhibiting protein translation of the target mRNA, and occasionally by inducing modification of histone and non-histone protein, and DNA methylation of promoter sites, which affects the expression of target genes. It is current dogma that regulation of miRNA preserves normal functioning of eukaryotic cells, whereas deregulation of miRNA may be associated with disease.

Case in point, two putative viral microRNA precursors (EBOV-pre-miR-1 and EBOV-pre-miR-2) and three putative mature microRNAs (EBOV-miR-1-5p, EBOV-miR-1-3p and EBOV-miR-2-3p) derived from the EBOV genome have been identified [202]. Data have also shown that certain proteins that are down-regulated in EBOV infection, such as tissue factor pathway inhibitor, dystroglycan1, caspase 8 FADD-like apoptosis regulator. Inhibition of the mi-RNAs hsa-miR-1246, hsa-miR-320a and hsa-miR-196b-5p can actually rescue the cell viability that was induced by EBOV glycoprotein [203]. Taken together these findings suggest a novel molecular basis for EBOV pathogenesis, and a new molecular therapeutic and preventive strategy for EBOV+ patients.

### Translational effectiveness

Recent developments in health care have witnessed the evolution of the original conceptualization of translational research into translational science in medicine, dentistry and nursing. Translational research, as originally defined by NIH, requires that sample biopsies obtained from individual patients be analyzed and characterized in the laboratory, and that the outcome of these studies be integrated in the clinical decision-making for treatment. Translational effectiveness, as later defined by AHRQ, defends that another major component of clinical decision-making must rest on obtaining, disseminating and utilizing the best available evidence in specific clinical settings [27]. Dissemination must be directed in various forms and formats to all of the stakeholders involved in the patient’s well-being - from the patients themselves, to the caregivers, the health-care team, and the patients’ friends and acquaintances. Stakeholders play a critical role in the dissemination process, be it person-to-person or via tele-medicine processes, in caring for patients with EBOV infection.

### Stakeholders in translational effectiveness

Stakeholders are important partners in the clinical decision-making process, particularly in the context of patient-centered healthcare homes and neighborhoods for optimizing patient-targeted interventions [27]. Stakeholders can include biologists, clinicians/clinical researchers, epidemiologists, health services researchers, patients, family members, caregivers, patient advocates, social workers, insurance carriers, and legal advisers, all striving to ensure multidimensional and multidisciplinary contributions to the multiplicity of patient-target endeavors in evidence-based, patient-centered and effectiveness-focused clinical decision-making [27]. Stakeholders contribute their skills and knowledge to synergistically share the responsibility of patient-targeted care. Active stakeholder engagement is critical to the success of patient-centered, effectiveness-focused and evidence-based intervention. Stakeholders in modern healthcare engage as:

- Primary stakeholders: those individuals ultimately and directly affected, either positively or negatively, by the healthcare outcomes (e.g., patients, the immediate family members, or caregivers of patients who cannot represent themselves).
- Key stakeholders: individuals who may or may not be primary stakeholders, but have a significant influence on the decision-making process (e.g., relatives, friends or caregivers empowered by a legal document or directive to make healthcare decisions on behalf of the patient).
- Secondary stakeholders: individuals indirectly affected by the outcomes, or indirectly involved in the patient’s care process.
- Allied stakeholders: individuals who are involved in the patient’s care, but are indirectly affected by the healthcare outcome (e.g., medical, dental, nursing and pharmacy staff, other hospital employees, insurance agents, legal staff and lawyers).

By contrast, incontrovertible evidence establishes that stakeholder engagement is rarely uniform: not all stakeholders have the same roles and degrees of involvement in the healthcare experience, and, as noted above, stakeholders may have very different perceptions of their responsibilities across diverse social and cultural environments. It follows that timely concerted research must be directed at the development and validation of novel analytical tools to establish the nature, level (or quantity), and quality of stakeholder participation in healthcare [27,204,205]. Case in point, the current spread of the Ebola epidemic might have been mitigated somewhat, or perhaps even impeded if proper stakeholder engagement procedures had been established and implemented.

Stakeholder engagement ought to be evaluated systematically in a process designed to identify and characterize the principal constructs and sub-constructs related to the stakeholders’ engagement in terms of their expected and perceived roles, involvement and function [204,205], including:

- stakeholders’ actions and position;
- ability and intent to influence implementation;
- motivation to participate; and
- capability to change and adapt as the care situation evolves, and calls for adaptive management)

Stakeholder engagement will play an important role in the psycho-emotional recovery of EBOV+ patients and patients afflicted with EVD. Further studies must be conducted to design a coherent set of contribution toward the patient well-being, such as increased health literacy through dissemination [206], psycho-emotional comfort, economic and societal support, etc., to validate stakeholders benefit [27,203-206].

### Telemedicine & eHealth

Telemedicine is an innovative medical technology system aimed at improving dissemination of the best available evidence and evidence-based revisions of clinical practice guideline, with the purpose of empowering clinicians, patients and all stakeholders toward clinically more sound and more informed decision-making. Telemedicine signifies connecting health care providers, patients and caregivers with expertise that is at great geographical distances (e.g., West Africa community Ebola treatment units with NIH health care system in Bethesda, MD) via electronic communication technologies. Telemedicine seeks to improve evidence-based, patient-centered and effectiveness-focused cutting edge medical intervention globally [27].

Implementation of telemedicine subsumes health information technologies (tele-care, electronic health records and digital medical chart, support systems, user-friendly multi-languages web-based packages and social media for special-needs patients, assistive information technologies [e.g. touchless keyboards and pointing devices to minimize cross-contamination], personal mobile phone apps, etc.), and as such can take several forms [207-209]:

- tele-care can provide health care and assistance to people who are physically less able (i.e. elderly, disabled), from patient’s own homes
- digital, paperless medical charts provide immediate access to patients’ medical records for efficient information exchange and consultation among medical specialists;
- support for diagnosis and clinical decision-making for treatment plan via computer based information and communication systems;
- web-based media for social support of patients and stakeholders.
- are helpful to patients by providing patients/caregivers information through social media accounts and web-based information.

Case in point, the *Réseau en Afrique Francophone pour la Télémédecine* (RAFT) network, was originally developed and established to connect Western European health care systems (e.g., France) to African communities (e.g., francophone Africa) in order to provide educational, clinical, and public health activities. Starting in the early 2000’s, educational programs were delivered on a weekly basis via video-lectures and digital communications and sharing to patient data and medical information for virtual continuing education and training, guided evidence-based clinical decision-making, and joint epidemiological research activities. The clinical and public health activities included web-based sharing of expertise for the management of infectious diseases and complex clinical cases, and for the implementation of clinical information systems in African hospitals. In 2010, the RAFT model was extended to the Altiplano in Bolivia and Nepal [209].

In brief, novel interventions involving the full utilization of telemedicine will be beneficial during the present Ebola pandemic primarily because it will connect all medical personnel across the world, from the *Médecins sans Frontières* in West African countries, to infectious disease specialists in Europe an the US through a seamless network of health care consultation, and joint progress toward the treatment of EBOV+ patients and their cure from EVD. Telemedicine is the best approach to bring much needed expertise to the most remote villages and communities where Ebola is rampant presently. Through telemedicine, hospital specialized in the diagnosis and the treatment of this disease, and state-of-the-art Ebola treatment units, such as in the US St. Patrick Hospital, Emory University Hospital, National Institutes of Health and Nebraska Medical Center, could connect digitally with any and all health care centers around the world for the purpose of sharing of medical data, information, consultation, and evaluation. More generally, the field of telemedicine can provide novel, timely and critical wireless, touchless and contamination-free instruments and devices – such as the devices recently introduced at certain airports in Europe and the US to monitor the passenger’s body temperature – whose measurements can be immediately recorded and transmitted digitally and wirelessly to national and international databases for health care information storage and exchange among hospitals, health care centers, WHO, CDC, and treating specialists.

From a bioethics perspective, and based on the RAFT experience, effective integration and optimum use of eHealth in emerging economies, such as West African countries presently assailed by the Ebola epidemic, must be articulated in a complex and interactive model of care. Novel interventions in telemedicine must coordinate the contextual health acre resources and infrastructure, the culture and anthropological nature of societal environment, the needs of stakeholders, and the fundamental understanding of the physiopathology, histopathology, cytopathology and molecular pathology of EBOV infection and the resulting EVD.

## Conclusion

The first cases of the current West African epidemic of Ebola virus disease, Ebola, were reported on the 22nd of March 2014. Seven months later, there were over 10,000 cases and close to 5,000 deaths, with about 20% EVD cases reported in health care workers, across several countries beyond the African continent, including the US and the European Union.

Signs and symptoms of Ebola usually begin suddenly with an influenza-like stage characterized by fatigue, fever, headaches, joint, muscle, and abdominal pain. Vomiting, diarrhea and loss of appetite are also common. Symptoms such as headache, encephalitis, meningitis, cerebral edema, and seizures commonly indicate CNS involvement. Less common symptoms include sore throat, chest pain, hiccups, shortness of breath and trouble swallowing. Skin manifestations include a maculopapular rash in about 50% of cases. The average time between contracting EBOV infection and the start of symptoms (incubation period) is eight to ten days, but it can vary between 2 and 21 days, or longer in some patients. Early symptoms of EVD mimic malaria, dengue fever or other tropical fevers, before the disease progresses to the bleeding phase. Specialized Ebola treatment units must be designated and established to prevent EBOV infection, and to control the spread of the disease. Intensive care in the developed countries includes maintaining blood volume and electrolytes homeostatic balance, and treating any collateral bacterial infections. Dialysis is required for kidney failure, and extracorporeal membrane oxygenation in the case of lung dysfunction. These interventions, however, are neither directed specifically to contain EBOV infection, nor available routinely in developing countries in Africa. It is timely and critical to rapidly increase Ebola treatment units, to expand their reach through telemedicine and eHealth protocols, and to develop and disseminate innovative treatment methods for communities urgently in need of support and assistance.

Cross-species transmissions, or “*when pathogens infecting one species ‘jump’ to infect another species*,” ([210], p. 117), are key to understanding global health in Africa’s forests and beyond. Most human EIDs result from those transmitted naturally between animals and humans through “*complex, dynamic, ongoing interactions between pathogens, people, other mammals, and forest ecologies*” ([210], p. 117). Recently, VHFs have developed into global pandemics and are now considered as threats to biosecurity. Sub-Saharan Africa provides a rich landscape for an understanding of the intersections between people, animals, pathogens, and their broader ecologies. Ebola outbreaks in this region provide a window to view “*disease transmission between animals and human beings, facilitated by the intensified circulation of people, capital, animals, pathogens, and technologies, (which) are a recurrent aspect of life on earth, often in highly uneven and unpredictable ways*.” ([210], p. 117) However, the reframing of biological threats in the rhetoric of biosecurity preparedness, with its emphasis on worst-case scenarios, often obscures the multi-causal dimensions, and the sociocultural and epidemiological consequences of EBOV infection and EVD pandemics that as often drive the new global public health agenda.

Considering that the Bracken Bat Cave outside of San Antonio, Texas holds the largest colony of bats worldwide, and that bats are the primary vector for EBOV, we ought to consider developing, in collaboration with Bat Conservation International, WHO, CDC and *Médecins sans Frontières*, a clinical immunotherapy program designed to develop, test and validate anti-EBOV vaccines for wildlife in general, and specifically targeted to bats. Given the biodiversity of these volant mammals, their distribution, patterns of migration and abundance, innovative approaches are necessary to control EBOV nationwide and internationally, including oral vaccination programs similar perhaps to those developed to control rabid bats and land mammal wildlife in developing countries [211,212].

However, the significant challenges of controlling the dynamic fluctuations of bats populations remain [213]. Recent data describing the determinants of viral richness in 15 species of African bats fully characterized for virus infection and bat phylogeny in Central and West Africa point to the need of more extensive knowledge of the variables that dictate the ecological, biological and evolutionary fragmentation of bat species in their geographical distribution and migration across the African continent and worldwide [214]. Taken together, these findings will emerge as critical to understand the zoonotic transmission of EBOV, and to inform novel interventions to prevent, control and cure EVD.

## Abbreviations

ACCN1: Amiloride-sensitive cation channel neuron-1
BBB: Blood brain barrier
CCR2: Chemokine receptor type 2
CDC: Center for Disease Control & Prevention
CDC5L: Cell division cycle 5-like protein
CEBPE: CCAAT/enhancer binding protein-ε
CI^95^: Confidence interval at 95%
CLDN3: Claudin 3
CNS: Central nervous system
CRHR2: Corticotropin releasing hormone receptor 2
CSF: Cerebrospinal fluid
DAMP: Damage-associated molecular patterns
DNA-PKCs: DNA-dependent serine/threonine protein kinase, catalytic subunit
EIDs: Emerging infectious diseases
EVD: Ebola virus disease
EBOV: Ebolavirus
FAM63A: Putative cytoskeletal protein
HMP19: Neuron-specific protein family member 2
ICAM1: Intercellular adhesion molecule-1
ICOS: Inducible costimulator
IFN-γ: Interferon-γ
IID: Interferon inhibitory domain
ILx: Interleukin-x
IL2RA: Interleukin-2 receptor-α, CD25
ILF2: Interleukin enhancer-binding factor 2
ILF3: Interleukin enhancer-binding factor 3
IP-10: Interferon gamma-induced protein 10
iNOS: inducible nitric oxide synthase
IRF3/7: Interferon regulatory factor 3 & 7
JAK/STAT: Janus kinase (JAK)/Signal transducer and activator of transcription (STAT)
LPS: Lipopolysaccharide
LTF: Lactoferrin
MBGV: Marburg virus
M-CSF: Macrophage colony-stimulating factor
MCP-1: Monocyte chemotactic protein 1 (also known as CCL2)
MIP-1α-β: macrophage inflammatory protein-1 α-β (also known as CCL3 & CCL4)
MIF: Macrophage migration inhibitory factor
NDUFA12: NADH dehydrogenase [ubiquinone] 1 alpha subcomplex subunit 12
PAI1: Plasminogen activator inhibitor 1
PAMP: Pathogen-associated molecular patterns
PPE: Personal protective equipment
PSMA1: Proteasome subunit alpha type-1
R0: Contact or exposure rates
RIGI: Retinoic acid inducible gene-I
RCHY1: Ring-finger and CHY-zinc finger domain-containing protein 1
RT-PCR: Reverse transcription polymerase chain reaction
RUVBL2: RuvB-like 2 (E. coli), second human homologue of the bacterial RuvB gene
sGP: soluble glycoprotein
sICAM1: soluble intracellular adhesion molecule 1
sVCAM1: soluble vascular cell adhesion molecule 1
ssGP: small soluble glycoprotein
SECIS: Seleno-cysteine insertion sequences
SLC9A7: Solute carrier family 9, subfamily A, member 7
SLC38A5: Sodium-coupled neutral amino acid transporter 5
STAT1: Signal transducers and activators of transcription-1
TBK1/IKKε: TANK-binding kinase 1/IkappaB kinase epsilon
TF: Tissue factor
TIM-1: T cell immunoglobulin and mucin domain 1
TNF-α: Tumor necrosis factor-α
Tregs: Regulatory T cell subpopulation (CD4/8 + CD25 + FoxP3+)
vGP1,2: viral glycoprotein 1,2
VHFs: Viral hemorrhagic fevers
VLP: Virus-like particle
VP: Viral protein
WHO: World Health Organization
